# Taxonomic revision of the Trapdoor spider genus *Eucteniza* Ausserer (Araneae, Mygalomorphae, Euctenizidae)

**DOI:** 10.3897/zookeys.356.6227

**Published:** 2013-11-28

**Authors:** Jason E. Bond, Rebecca L. Godwin

**Affiliations:** 1 Department of Biological Sciences, Auburn University Museum of Natural History, Alabama, USA

**Keywords:** Biodiversity, new species, spider taxonomy, *Eucteniza*, Euctenizidae, Mygalomorphae

## Abstract

The mygalomorph spider genus *Eucteniza* Ausserer, 1875 comprises 15 nominal species known only from the southwestern United States (Texas) and Mexico (Northern, Central, and the Baja Peninsula). *Eucteniza atoyacensis* Bond & Opell, 2002 is considered a nomen dubium; *E. rex* (Chamberlin, 1940) and *E. stolida* (Gertsch & Mulaik, 1940) are both considered junior synonyms of *E. relata* (O.P.-Cambridge, 1895). Twelve new species are described: *E. caprica*, *E. coylei*, *E. diablo*, *E. cabowabo*, *E. huasteca*, *E. zapatista*, *E. chichimeca*, *E. ronnewtoni*, *E. hidalgo*, *E. golondrina*, *E. panchovillai* and *E. rosalia*.

## Introduction

**Figure 1.**
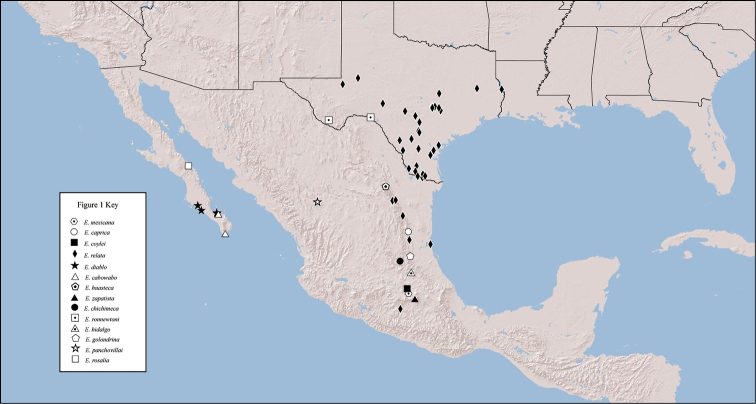
Distribution of known *Eucteniza* species.

The trapdoor spider *Eucteniza* Ausserer, 1875 (subfamily Euctenizinae) is the most distinguishable of the genera currently placed in the recently recognized North American spider family Euctenizidae (see Bond et al. 2012). Known species have a unique mating clasper (modifications of the first walking leg) that comprises a mid-ventral tibial megaspine with similar modifications to the second walking leg. Members of the genus also have a lightly sclerotized, “soft”, dorsal posterior aspect of the carapace that is very noticeable in live and preserved specimens. The distribution of the genus is largely restricted to the US state of Texas and northern/central Mexico (Fig. 1). As such, the habitat is predominantly low elevation, desert and tropical dry forest. The relatively few female specimens that have been hand collected were recovered from silk-lined burrows, reminiscent of *Ummidia* Thorell, 1875 (see Bond and Coyle 1995) – they have a thick silk lining and heavy “cork”-like trapdoor.

Phylogenetic placement of the genus has been historically problematic. Until recently, *Eucteniza* was the type genus for the cyrtaucheniid subfamily Euctenizinae (see Raven 1985, Bond and Hedin 2006). Morphological cladistic analyses (Bond and Opell 2002) and subsequent molecular phylogenetic analyses (Bond and Hedin 2006, Hedin and Bond 2006) clearly demonstrated Cyrtaucheniidae to be polyphyletic; imprecisely referenced as “possibly paraphyletic” by Platnick (2013) in the World Spider Catalog citing an older now superseded study by Goloboff (1993). The most recent multi-gene molecular phylogenetic analysis of the mygalomorph families by Bond et al. (2012) clearly supported a monophyletic Euctenizinae, which was sister to idiopids, and a polyphyletic Cyrtaucheniidae. Consequently the subfamily was elevated to family status and currently comprises seven genera: *Apomastus* Bond & Opell, 2002, *Aptostichus* Simon, 1891, *Entychides* Simon, 1888, *Eucteniza* Ausserer, 1875, *Myrmekiaphila* Atkinson, 1886, *Neoapachella* Bond & Opell, 2002, and *Promyrmekiaphila* Schenkel, 1950. Upon relimitation of the subfamily Euctenizinae by Bond et al. (2012), *Eucteniza* is considered more closely related to *Promyrmekiaphila*, *Neoapachella*, *Entychides*, and a potentially new genus from California. Although some analyses render *Entychides* paraphyletic with respect to *Eucteniza*, members of these two genera are very distinct morphologically and thus *Entychides* paraphyly is likely an artifact of limited sampling.

Like other euctenizid genera, the taxonomic history of *Eucteniza* is brief but does include a number of generic level synonyms. First described by Ausserer (1875), subsequent species were proposed but were all placed into other genera later considered by Bond and Opell (2002) to be junior synonyms. The North American species, *Eucteniza rex* (Chamberlin, 1940) and *Eucteniza stolida* (Gertsch & Mulaik, 1940) were originally described as *Astrosoga* Chamberlin, 1940 taxa, a genus proposed by Chamberlin (1940) as closely allied with *Myrmekiaphila*. *Flavila* O.P.-Cambridge, 1895 and *Enrico* O.P.-Cambridge, 1895 were earlier names proposed for Mexican species; *Flavila* was shortly thereafter recognized as a junior synonym of *Eucteniza* by F.O.P.-Cambridge (1897) with the synonymy of *Enrico* following much later (Bond and Opell 2002).

We present here the first species level taxonomic revision of the genus *Eucteniza*; this is the tenth paper in a series of taxonomic revisions and reviews and phylogenetic treatments undertaken by the first author (JEB) seeking to resolve the taxonomy of the North American euctenizid genera and species (Bond and Opell 2002, Bond 2004, Bond and Hedin 2006, Bond and Platnick 2007, Stockman and Bond 2008, Bond and Stockman 2008, Bailey et al. 2010, Bond et al. 2012, Bond 2012). Unfortunately, *Eucteniza* specimens are rare in collections and difficult to collect (Bond pers. obs.) thus with the exception of *Eucteniza relata* (distributed widely throughout Texas and Northern Mexico), most of the species described herein are based on relatively little material and most are known only from male specimens. Moreover, a number of species, to include the type species for the genus, were originally described from juvenile specimens; one such species, *Eucteniza atoyacensis* Bond & Opell, 2002 is considered herein a nomen dubium. Given the paucity of material it is our hope that this work will catalyze interest in the genus and facilitate future studies.

**Species concept applied**: Species were delineated using a traditional morphological species concept wherein species are defined as those populations (or groups of populations) that represent qualitative differences in phenotype that differ in a discrete manner from other populations or groups.

## Materials and methods, abbreviations

The following institutional and quantitative morphological abbreviations used in this paper are defined as follows:

### Institutional

AMNH (American Museum of Natural History; New York, New York), AUMNH (Auburn University Museum of Natural History; Auburn, Alabama), BMNH (British Museum of Natural History; London), CAS (California Academy of Sciences; San Francisco, California).

### Quantitative Morphological

These features are explicitly defined and illustrated in Bond (2012)

ANTd number of teeth on the anterior margin of the female cheliceral fang furrow

Cl, Cw carapace length and width. Carapace length taken along the midline dorsal most posterior position to the anterior front edge of the carapace (chelicerae are not included in length). Carapace width taken at the widest point

LBl, LBw labium length and width taken from the longest and widest points, respectively

PTl, PTw male palpal tibia length and width

Bl palpal bulb length from embolus tip to the bulb base, taken in the ventral plane at its longest point

PTLs, TBs number of female prolateral patella and tibial spines leg III

STRl, STRw sternum length and width. Sternum length from the base of the labium to its most posterior point. Width taken across the widest point, usually between legs II and III

TSrd, TSp, TSr number of tibia I spines on the distal most retrolateral, prolateral, and midline retrolateral positions

### Measurement, characterization, and illustration of morphological features

Unique voucher numbers were assigned to all specimens (alphanumeric designations beginning with EU, MY, or UMM); these data were added to each vial and can be used to cross-reference all images, measurements, and locality data. All measurements are given in millimeters and were made with a Leica M165c dissecting microscope equipped with the Leica Analysis Suite Software. Lengths of leg articles were taken from the mid – proximal point of articulation to the mid – distal point of the article (*sensu* Bond 2012, figures 11–16). Leg I and Leg IV article measurements are listed in the species descriptions in the following order: femur, patella, tibia, metatarsus, tarsus. Carapace and leg coloration are described semi-quantitatively using Munsell® Color Charts (Windsor, NY) and are given using the color name and color notation (hue value/chroma).

Mating clasper line drawings were first recorded as digital images and then traced as vector drawing objects using Adobe Illustrator (Adobe Systems Inc.). Digital images of specimens were made using a Visionary Digital Imaging System (Visionary Digital^TM^, Richmond, VA) where images were recorded at multiple focal planes and then assembled into a single focused image using the computer program Helicon Focus (Helicon Soft, Ltd., Ukraine). The female genital region was removed from the abdominal wall and tissues dissolved using trypsin; spermathecae were examined and photographed in the manner described above. Following Bond (2012) and Bond and Taylor (2013) habitus illustrations were constructed from whole body images that were bisected, copied, and reflected in Adobe Photoshop (Adobe Systems, Inc.) to produce a roughly symmetrical image; the actual raw image on which the habitus illustration is based has been deposited in Morphbank and its record number noted in the figure legend (value in square [ ] brackets). Unless otherwise stated, scale bars = 1.0 mm.

### Locality data and georeferencing

Latitude and longitude for all collecting localities were recorded in the field using a Garmin® Global Positioning System receiver (Garmin International Ltd., Olathe, KS) using WGS84 map datum. For previously collected specimens (e.g., loaned museum specimens) locality data were georeferenced by hand by finding the approximate locality using Google Earth (WGS84 datum). A distribution map was constructed using ArcGIS using NAD83 map datum. Specimens without latitude and longitude data were georeferenced as described by Bond (2012). Precision for each georeferenced point is annotated as a superscript in each material examined section of the species’ taxonomy using the confidence value scheme employed by Murphey et al. (2004): 1 = exact coordinates given; 2 = amended exact coordinates (i.e., exact coordinates given but were emended on validation); 3 = public land survey system; 4 = within 1km radius; 5 = within 5km radius; 6 = within 10km radius; 7 = to county or > 10km; 8 = to state; 9 = to project region. Latitude and longitude are recorded to the 4^th^ decimal place as an indication of the precision in the point assigned by us (i.e., where we have assigned the locality place-holder for the specimen in question), not precision in the recording of the value or to specify the exact point of collection. Detailed locality and associated GIS data as supplemental data files in spreadsheet and KML file format can be downloaded online from the Dryad Data Repository at doi: 10.5061/dryad.6dc14.

### Data resources

The data underpinning the analysis reported in this paper (see below) were deposited on 18 November 2013 in the Dryad Data Repository at doi: 10.5061/dryad.6dc14 and at GBIF, the Global Biodiversity Information Facility, http://ipt.pensoft.net/ipt/resource.do?r=eucteniza_data. Images associated with species descriptions have been deposited in Morphbank (http://www.morphbank.net); Morphbank image record numbers are noted in brackets by each figure in the figure legend.

## Taxonomy

### Family Euctenizidae

Raven, 1985

#### Type genus.

*Eucteniza* Ausserer, 1875

### Subfamily Euctenizinae

Raven, 1985

http://zoobank.org/C27FB688-5D8E-4E77-ABCC-FD108DC4C22D

#### Included genera.

*Entychides* Simon, 1888; *Eucteniza* Ausserer, 1875; *Neoapachella* Bond & Opell, 2002; *Promyrmekiaphila* Schenkel, 1950.

### Genus Eucteniza

Ausserer, 1875

http://zoobank.org/F1037BA5-A80D-47F9-8F15-6937D62F89E4

http://species-id.net/wiki/Eucteniza

Figs 1 –7

Eucteniza Ausserer, 1875: 149 (type species by monotypy *Eucteniza mexicana* juvenile holotype from Mexico, deposited in BMNH, examined). – E. Simon 1892: 110. – F.O.P.-Cambridge 1897: 12. – Bond and Opell 2002.
Flavila O.P.-Cambridge, 1895: 156 (type species by monotypy *Flavila relatus* O.P.-Cambridge, male holotype from Mexico, Amula in Guerrero, deposited in the BMNH, examined). – synonymized by F.O.P.-Cambridge 1897: 13.
Enrico O.P.-Cambridge, 1895: 157 (type species by monotypy *Enrico mexicanus* juvenile holotype from Mexico, Atoyac, Veracruz, deposited in BMNH, examined). – F.O.P.-Cambridge 1897: 12. – E. Simon 1903: 899. – synonymized by Bond and Opell 2002.
Astrosoga Chamberlin, 1940: 5 (type species by monotypy *Astrosoga rex* male holotype from Kingsville, Texas, deposited in AMNH, examined). – Chamberlin and Ivie 1945: 556. – synonymized by Bond and Opell 2002.

#### Diagnosis.

*Eucteniza* males can be recognized by the presence of 1-2 mid-ventral megaspines on the tibia of both legs I and II (Figs 8–10). Such mating clasper spination configuration is similar to that of *Neoapachella* males for leg I but are absent on leg II. Females can be distinguished from all other euctenizid genera by having what appears to be a bi-dentate cheliceral furrow and a rastellum positioned on a moderate to high rastellar mound, whereas other genera have a single row of promarginal teeth and a small patch of denticles and lack a distinct rastellar mound. Additional *Eucteniza* autapomorphies include a patella IV spine patch and a weakly sclerotized posterior carapace margin.

#### General description.

**Figures 2–7.**
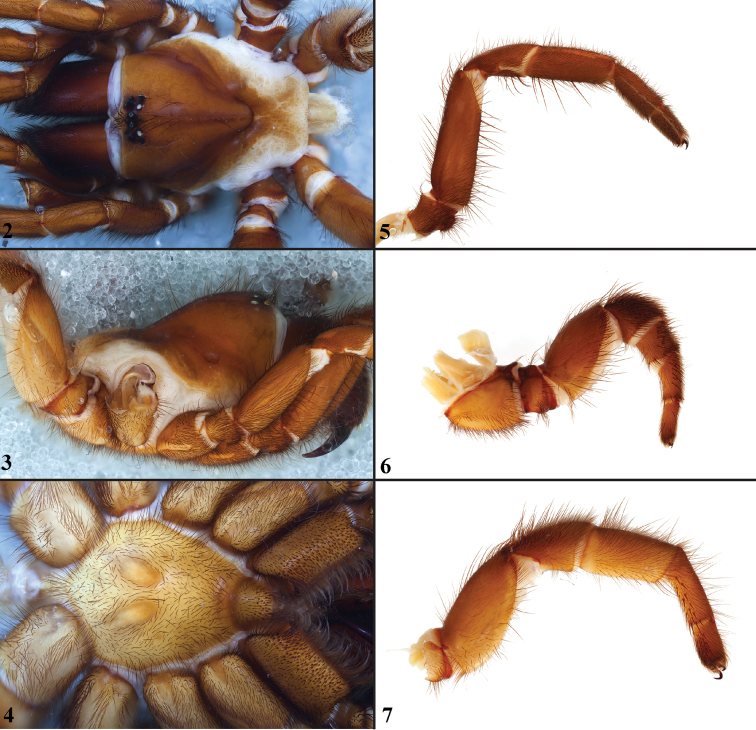
Standard light microscopy views of female *Eucteniza relata* (O.P.-Cambridge, 1895) from Hidalgo Co., TX. **2** dorsal view **3** side view **4** ventral view of sternum, labium and palpal endites **5** right leg I, retrolateral view **6** left leg III, prolateral view **7** left leg IV, prolateral view.

Small to large sized trapdoor spiders. Cephalothorax longer than wide, sloping posteriorly, lacking pubescence in most species (Fig. 2). Posterior third of carapace very lightly sclerotized (Figs 2, 23, 24). Thoracic groove intermediate to wide, procurved (Fig. 2) and deep. Eyes not on a tubercle (Fig. 3). AME, PME subequal diameter. Posterior eye row slightly recurved, anterior eye row slightly porcurved (Fig. 2). Caput moderately high (Fig. 3). Carapace of ethanol preserved specimens appears most often reddish-brown, sometimes lighter. The coloration of living spiders tends to be a darker brown, however there is considerable variation in the intensity of coloration. Male coloration in most specimens is dark reddish-brown. Abdominal coloration light to dark brown, sometimes with dark mid dorsal blotch.

Sternum wider posteriorly, tapering anteriorly (Fig. 4). Posterior sigilla large and positioned mid-posteriorly nearly contiguous. Anterior margin of sigilla lacks rounded margin. Palpal endites longer than wide with numerous cuspules (Fig. 4). Labium wider than long, with numerous cuspules (Fig. 4). Chelicerae dark brown. Rastellum consists of numerous spines borne on a distinctive mound. Fangs of intermediate length and thickness. Cheliceral promargin with row of very large teeth; retromargin row comprises distinct row of large teeth interspersed with denticles.

Apical PLS article short, digitiform. Spinnerets mostly with pumpkiniform spigots with several articulated spigots interspersed on apical and median articles of PLS and the PMS (Bond and Opell 2002, fig. 3E). Two to three large, articulated spigots on apical most aspect of the PLS. PMS article robust. See Bond and Opell (2002) for more detailed descriptions of spigot types.

Anterior leg articles slender relative to posterior. Tarsi short and robust (Figs 5–6). Female scopulae long, dense, asymmetrical, extending full length of tarsus, metatarsus and half length of tibia on anterior legs; posterior legs lack distinct scopulae. Male tarsi I and II with short sparse scopulae that are restricted to the ventral surface. Basal palpal tooth and STC I – IV basal tooth elongate and bifid. STC IV with 5 or more teeth. Female anterior legs with very few ventral spines (Fig. 5). Prolateral surface of female patella III and IV covered in numerous thick short spines (Fig. 6). Preening comb on metatarsus IV absent; metatarsus, tarsus IV with ventral spines (Fig. 7). Tarsal trichobothria arranged in a wide band with interspersed setae. Spermathecae generally comprise a simple unbranched bulb that lacks an elongate base.

Male mating clasper morphology is distinctive. Tibia legs I & II swollen mid-ventrally in most species, bearing 1-2 large spines; prolateral aspect with a small to large patch of smaller, thickened, short spines. Metatarsus of leg I lacks excavation and spur. Palpal bulb simple, with spherical base, planar distally near origin of embolus. Palpal cymbium lacks dorsal spines (Fig. 11).

**Figures 8–12.**
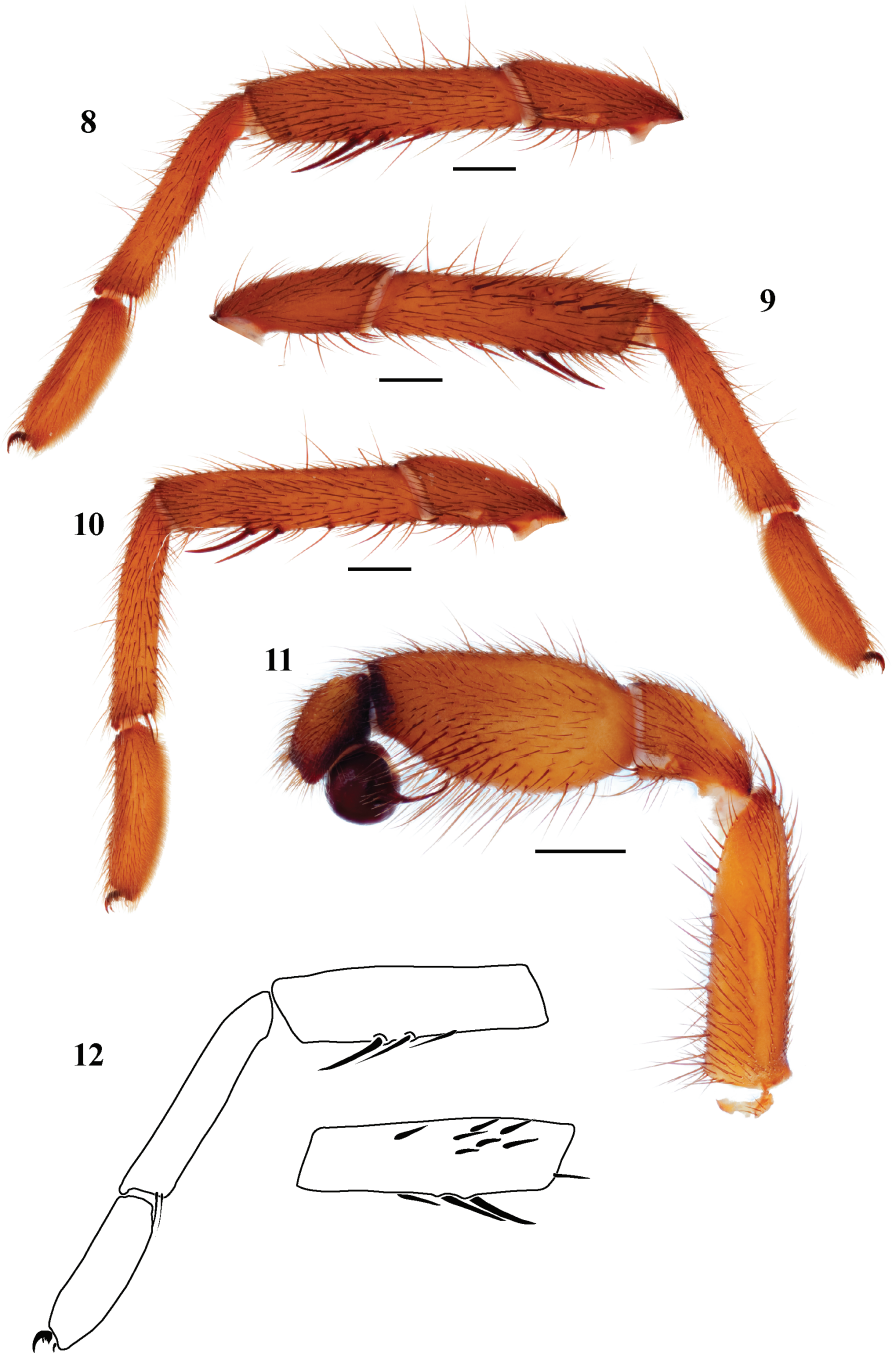
*Eucteniza mexicana* Ausserer, 1875, male exemplar specimen from Mexico Distrito Federal, Mexico. **8** retrolateral aspect, leg I [831980] **9** prolateral aspect, leg I [831976] **10** retrolateral aspect, leg II [831982] **11** retrolateral aspect, pedipalp [831984] **12** line drawings, leg I retrolateral and prolateral (tibia) aspects.

#### Distribution.

Distributed primarily throughout central Mexico and Baja California (Fig. 1) with an extension northward into Texas (United States).

#### Key to males

*Note*: as discussed by Bond (2012) keys to many mygalomorph taxa are sometimes far from optimal and thus one should not rely too heavily on species determinations using this key. Instead, knowledge of where specimen was collected and comparison to description and illustrations will likely prove more useful.

**Table:** 

1	Tarsus swollen mid-ventrally, width wider than metatarsus (Fig. 8)	*Eucteniza mexicana*
–	Tarsus width subequal to metatarsus width	2
2	Tibia I swollen dorsally, behind tibia I metatarsus junction (Fig. 53)	*Eucteniza chichimeca*
–	Tibia I not swollen dorsally behind tibia I metatarsus junction	3
3	Ventral tibial megaspines borne on distinct apophysis (Fig. 31)	4
–	Ventral tibial megaspines not borne on a distinct apophysis	5
4	Tarsus I with short dorsal spines, tarsus III curved (Figs 31, 35)	*Eucteniza diablo*
–	Tarsus I lacks short spines, palpal tibia retrolateral surface with extensive spine patch (Figs 48, 51, 52)	*Eucteniza zapatista*
5	Metatarsus I with ventral microspines and subdorsal row of spines on prolateral surface tibia II (Figs 64, 66)	*Eucteniza hidalgo*
–	Metatarsus I lacking ventral microspines, and leg II prolateral spines on tibia	6
6	Palpal tibia with row of retrolateral spines at distal edge and metatarsus I with patch of distal ventral spines (Figs 69, 72, 73)	*Eucteniza golondrina*
–	Palpal tibia without row of retrolateral spines at distal edge; metatarsus lacks distinct ventral spines (numerous)	7
7	Very small (Cl < 3.5mm); very pale in coloration	*Eucteniza huasteca*
–	Typically larger in size (Cl > 4.00mm); darker in color	8
8	Leg I metatarsus as long as or subequal in length to tibia; tibia slender with thin ventral megaspines (Fig. 37)	*Eucteniza cabowabo*
–	Leg I tibia shorter than metatarsus, ventral megaspines typically thicker and tibia not slender (usually swollen mid-ventrally)	9
9	Leg I prolateral tibial spines are longer in length	10
–	Leg I prolateral tibial spines are shorter in length	11
10	Leg I prolateral tibial spines fewer (10), longer in length, and thinner; spider paler in color (Fig. 14)	*Eucteniza caprica*
–	Leg I with more prolateral tibial spines (14) which are relatively shorter in length and stouter (Fig. 19)	*Eucteniza coylei*
11	Tibia I with very few prolateral spines (< 4) with few (1) spines situated distally (Fig. 60)	*Eucteniza ronnewtoni*
–	Tibia I with larger number of prolateral spines (>3) with spines more evenly distributed distally to proximally (Fig. 26)	*Eucteniza relata*

##### Nomen dubium

***Eucteniza atoyacensis*** Bond & Opell, 2002. Replacement name: *Enrico mexicanus* (O.P.-Cambridge, 1895) = *Eucteniza atoyacensis*. Holotype specimen is a juvenile and thus no known specimens or species can be unambiguously attributed to this name at this time.

### Eucteniza mexicana

Ausserer, 1875

http://species-id.net/wiki/Eucteniza_mexicana

Figs 1 , 8–12

Eucteniza mexicana Ausserer, 1875: 149; juvenile holotype from Mexico, deposited in BMNH, examined. – E. Simon 1892: 110. – F.O.P.-Cambridge 1897: 12. – Bond and Opell 2002.

#### Exemplar material.

Male exemplar (EU008) from Mexico Distrito Federal, Mexico, 19.4327, -99.1347^8^, elev. 2249m, coll. J. Honey, deposited in AMNH.

#### Diagnosis.

*Eucteniza mexicana* is similar to *Eucteniza coylei* and *Eucteniza caprica* in appearance but has more stout tarsi on leg I (Figs 8–10, 12) and fewer prolateral leg I tibial spines that are short and thick; prolateral tibial spines on the other two species are longer and thinner.

#### Description of male exemplar.

*Specimen preparation and condition*. Specimen preserved in 70% EtOH. Pedipalp, legs I, II removed, stored in vial with specimen. Color faded. *General coloration*. Carapace dark reddish brown 2.5YR 2.5/4. Abdomen very dark brown 7.5YR 2.5/3. *Cephalothorax*. Carapace 6.707 long, 5.548 wide, sparsely setose, pars cephalica slightly elevated. Fringe sparse. Foveal groove deep, procurved. Tubercle absent. AER slightly procurved. PER slightly recurved. AME slightly larger in diameter than PME. Sternum moderately setose, STRl 3.825, STRw 3.401. Posterior sternal sigilla very large, not contiguous, medial pair of anterior sigilla moderate in size and inset, anterior pair small and marginal. Chelicerae with anterior tooth row comprising 7 large teeth, posterior margin with single row of 9 small teeth. Palpal endites with numerous cuspules across endite face, labium with 7-9 cuspules, LBw 1.086, LBl 0.613. Rastellum consists of 7 small spines on a small mound. *Abdomen*. Moderately setose. *Legs*. Leg I: 6.087, 3.050, 4.303, 3.920, 2.572; leg IV: 6.341, 2.824, 4.771, 5.836, 3.508. Dense scopulae on legs I-II. Tarsus I with wide band of 14 trichobothria. Leg I spination pattern (Figs 8, 9, 12); TSp 8, TSr 0, TSrd 0; leg II with two ventral thin megaspines. *Pedipalp*. PTw 1.570, PTl 2.908, Bl 1.430. Embolus arises sharply from bulb and tapers quickly, geniculate at tip (Fig. 11).

#### Variation.

Known only from the exemplar specimen and juvenile holotype

#### Distribution.

Highly imprecise, Mexico; exemplar specimen from Mexico Distrito Federal (Fig. 1).

### Eucteniza caprica sp. n.

http://zoobank.org/2664B285-A666-43EB-9735-62FF425E1026

http://species-id.net/wiki/Eucteniza_caprica

**Figures 13–17.**
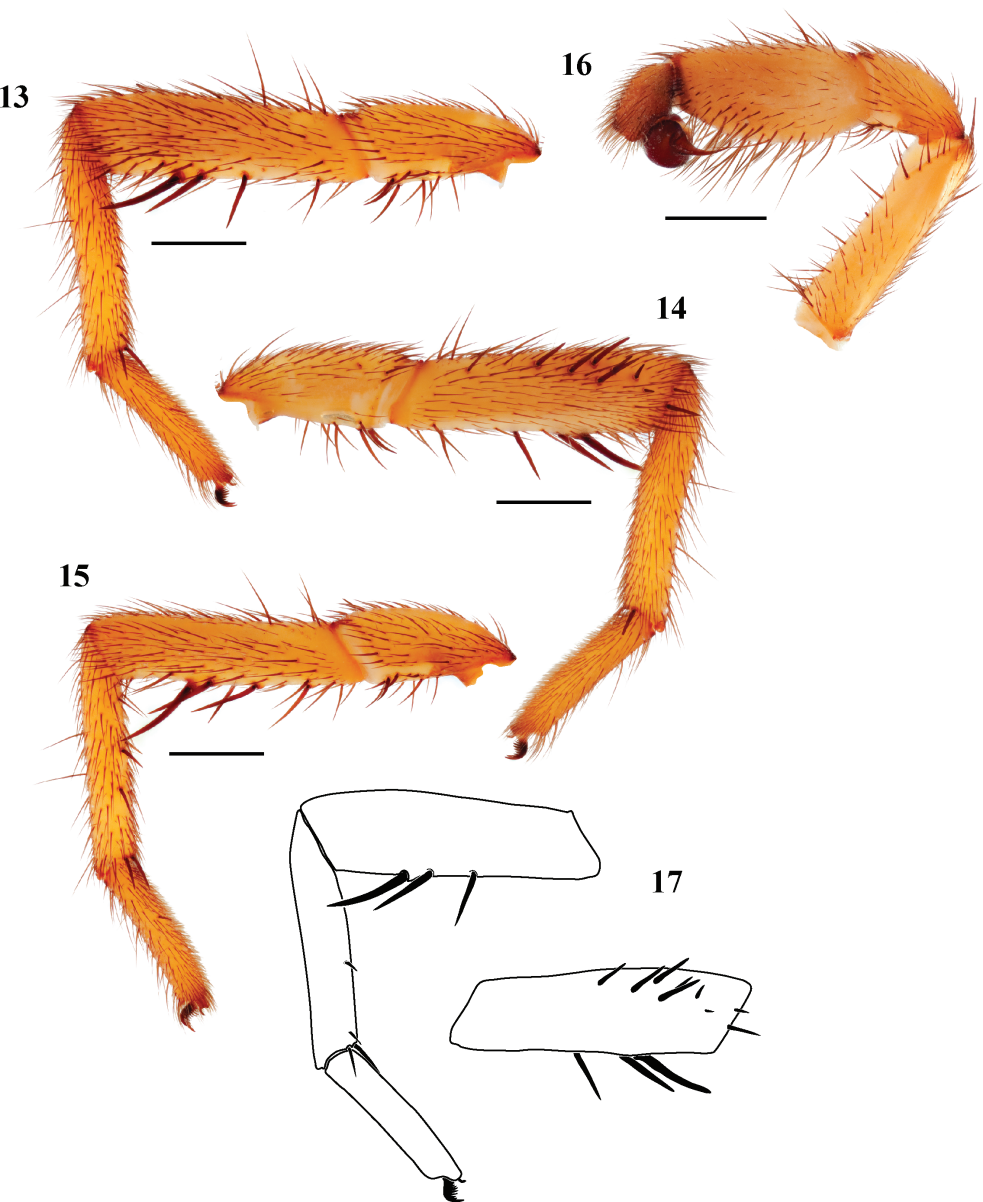
*Eucteniza caprica* sp. n. male holotype specimen from Tamaulipas, Mexico. **13** retrolateral aspect, leg I [832084] **14** prolateral aspect, leg I [832080] **15** retrolateral aspect, leg II [832086] **16** retrolateral aspect, pedipalp [832088] **17** line drawings, leg I retrolateral and prolateral (tibia) aspects.

The ‘Caprica-Six Trapdoor Spider’ Figs 1 , 13–17

#### Type material.

Male holotype (EU106) from Tamaulipas, Mexico, 23.0303, -99.1478^5^, elev. 335m, coll. G. Farias 13.iii.1972, deposited in AMNH.

#### Etymology.

The specific epithet is a noun taken in apposition and is in reference to the humanoid cylon model Caprica 6, portrayed by Tricia Helfer in the remake of the science fiction series Battlestar Galactica.

#### Diagnosis.

*Eucteniza caprica* is similar to *Eucteniza mexicana* in appearance but is smaller in size and lighter in coloration, leg I tarsi are not nearly as stout (Figs 13–15, 17).

#### Description of male holotype.

*Specimen preparation and condition*. Specimen preserved in 70% EtOH. Pedipalp, legs I, II removed, stored in vial with specimen. Color faded. *General coloration*. Carapace strong brown 7.5YR 5/6. Abdomen light yellowish brown 10YR 6/4. *Cephalothorax*. Carapace 4.712 long, 4.041 wide, sparsely setose, pars cephalica slightly elevated. Fringe sparse with 2-3 heavy setae at posterior corners. Foveal groove deep, procurved. Tubercle absent. AER slightly procurved. PER slightly recurved. AME slightly larger in diameter than PME. Sternum moderately setose, STRl 2.615, STRw 2.301. Posterior sternal sigilla very large, elongate, not contiguous, anterior sigilla pair not visible. Chelicerae with anterior tooth row comprising 6 large teeth, posterior margin with single row of 3 small teeth. Palpal endites lacking cuspules across endite face, labium lacking cuspules, LBw 0.892, LBl 0.548. Rastellum consists of 5 small spines not on a mound. *Abdomen*. Moderately setose. *Legs*. Leg I: 4.583, 2.141, 2.930, 2.872, 1.862; leg IV: 5.227, 2.113, 4.124, 4.557, 2.763. Light scopulae on legs I-II. Tarsus I with wide band of 15 trichobothria. Leg I spination pattern (Figs 13, 14, 17); TSp 10, TSr 0, TSrd 0. *Pedipalp*. PTw 0.985, PTl 1.942, Bl 0.932. Embolus arises sharply from bulb and tapers quickly, slightly flared at tip (Fig. 16).

#### Variation.

Known only from the male holotype specimen.

#### Distribution.

Known only from the type locality, Tamaulipas, Mexico (Fig. 1).

### Eucteniza coylei sp. n.

http://zoobank.org/F183B402-2346-4296-8DFD-6D1324216110

http://species-id.net/wiki/Eucteniza_coylei

**Figures 18–22.**
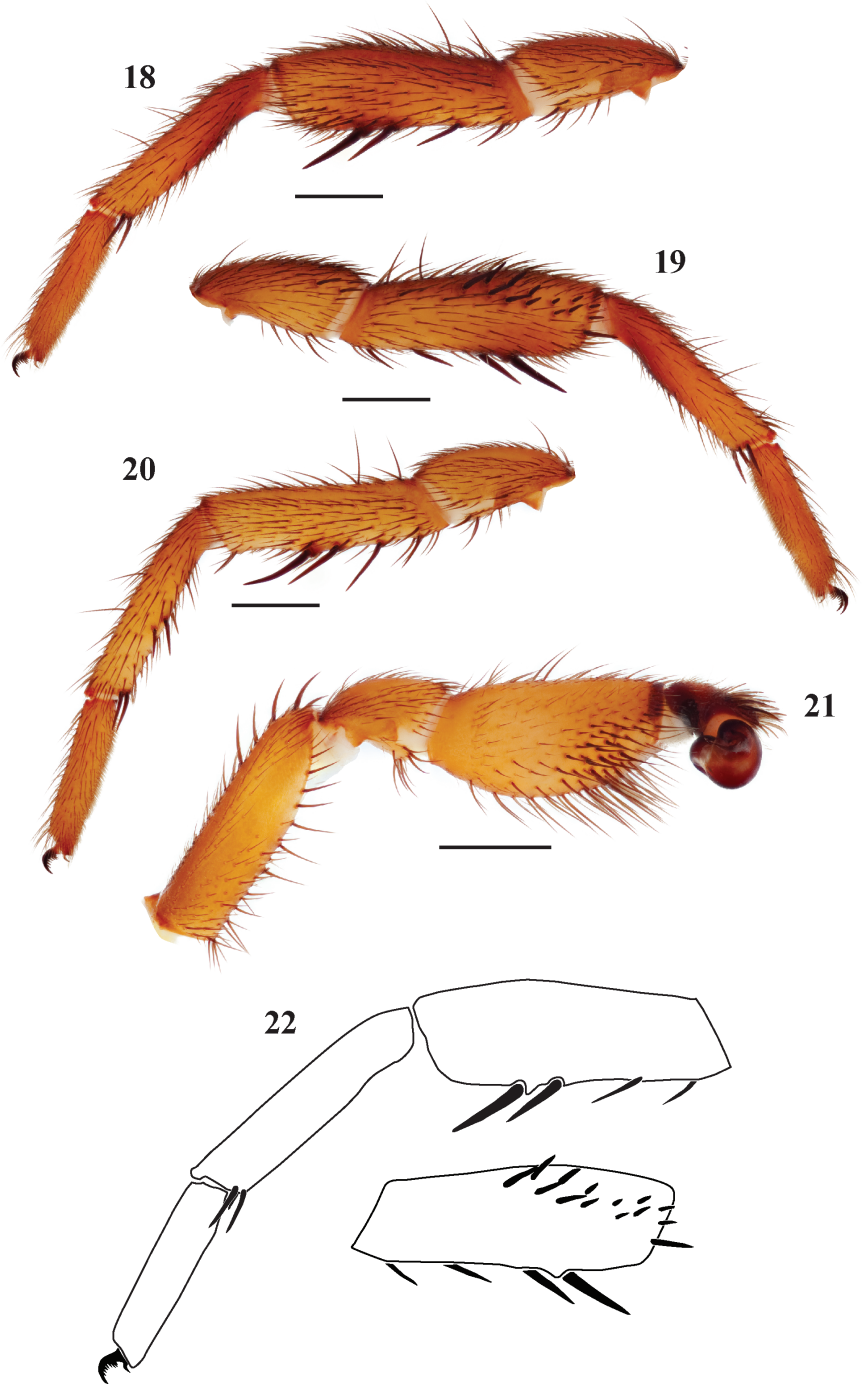
*Eucteniza coylei* sp. n. male holotype specimen from Morelos, Mexico. **18** retrolateral aspect, leg I [831990] **19** prolateral aspect, leg I [831986] **20** retrolateral aspect, leg II [831992] **21** retrolateral aspect, pedipalp [831994] **22** line drawings, leg I retrolateral and prolateral (tibia) aspects.

‘Coyle’s Trapdoor Spider’ Figs 1 , 18–22

#### Type material.

Male holotype (EU009), from Morelos, Mexico, 0.8km W Tepozitlán, Rt 1150 on rd to Ocotepec, 18.9889, -99.1116^8^, 1822m, coll. F. Coyle 10.vi.1982. Male holotype deposited in AMNH.

#### Etymology.

The specific epithet is a patronym in honor of arachnologist Fred Coyle who collected the type specimen.

#### Diagnosis.

*Eucteniza* is similar in appearance to *Eucteniza mexicana* but has thinner tarsi and short tibia I (Figs 19, 22) prolateral spines that are concentrated distally whereas *Eucteniza mexicana* lacks short distal spines on tibia I.

#### Description of male holotype.

*Specimen preparation and condition*. Specimen preserved in 70% EtOH. Pedipalp, legs I, II removed and stored in vial with specimen. Color faded. *General coloration*. Carapace dark red 2.5YR 3/6. Abdomen reddish black 2.5YR 2.5/1. *Cephalothorax*. Carapace 4.75 long, 4.00 wide, glabrous, pars cephalica moderately elevated. Fringe of sparse black setae. Foveal groove deep, procurved. AER slightly procurved, PER straight. AME and PME subequal. Sternum moderately setose, STRl 2.83, STRw 2.76. Posterior sternal sigilla large, not contiguous, anterior sigilla pairs small and marginal. Chelicerae with anterior row comprising 6 large teeth, posterior margin with a patch of approximately 12 small teeth. Palpal endites and labium without cuspules, LBw 0.79, LBl 0.58. Rastellum consists of 8 spines. *Abdomen*. Setose, thin, fine black setae. *Legs*. Leg I 4.20, 2.20, 2.88, 2.56, 1.84; leg IV: 4.30, 2.00, 3.75, 3.80, 2.25. Light scopulae on legs I, II. Tarsus I with 10 widely spaced trichobothria. Leg I spination pattern (Figs 19, 20, 22); TSp 14, TSr 0, TSrd 0. *Pedipalp*. PTw 1.58, PTl 2.00, Bl 1.45. Embolus relatively short, flared at tip (Fig. 21).

#### Variation.

Known only from the type specimen.

#### Description of female.

Known only from the male holotype specimen.

#### Distribution.

Known from the type locality in Morelos, Mexico (Fig. 1).

### Eucteniza relata

(O.P.-Cambridge, 1895)

http://species-id.net/wiki/Eucteniza_relata

‘The Southwestern Trapdoor Spider’ Figs 1 , 23 –30

Flavila relatus O.P.-Cambridge, 1895: 156; male holotype from Mexico, Amula in Guerrero, deposited in the BMNH, examined in 2002. – F.O.P.-Cambridge 1897: 13.
Eucteniza rex (Chamberlin, 1940): 5; male holotype from Kingsville, Texas, deposited in AMNH, examined. – Chamberlin and Ivie 1945: 556. – Bond and Opell 2002. **syn. n.**
Eucteniza stolida (Gertsch & Mulaik, 1940): 310; female holotype from Austin, Texas, deposited in AMNH, examined. – Bond and Opell 2002. **syn. n.**

#### Diagnosis.

*Eucteniza relata* mating clasper morphology comprises 2 large, tightly grouped megaspines on the mid-ventral aspect of tibia I; few (4) to many prolateral distal spines (21); tibia I medially swollen (Figs 25, 26, 29).

**Figures 23, 24.**
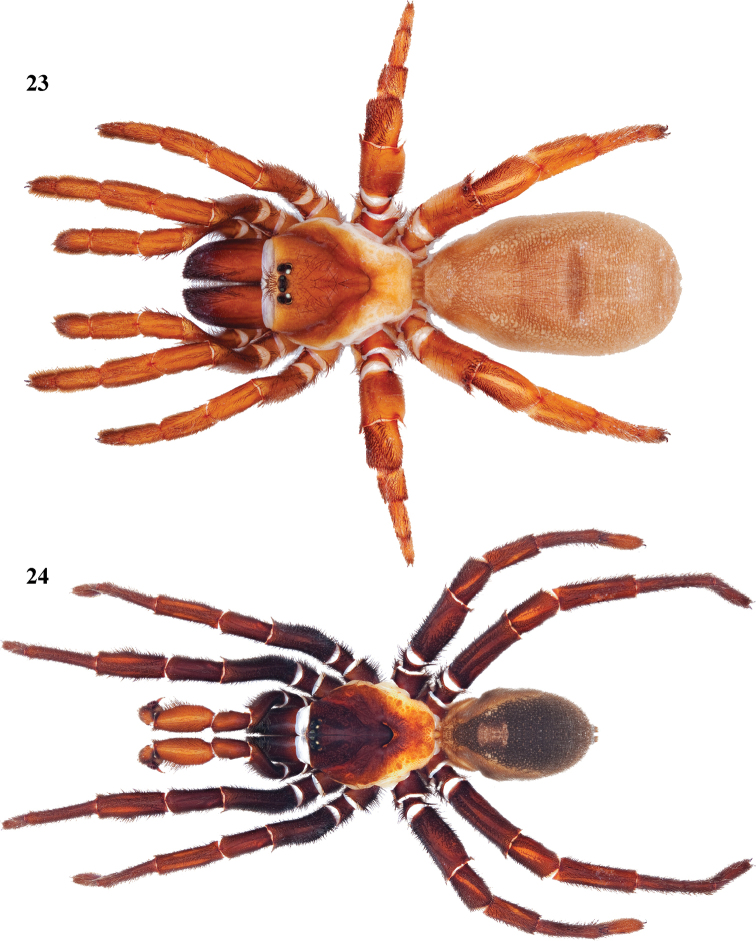
*Eucteniza relata* (O.P.-Cambridge, 1895) from Kingsville, Texas **23** female habitus illustration [832092] **24** male habitus illustration [832056].

**Figures 25–30.**
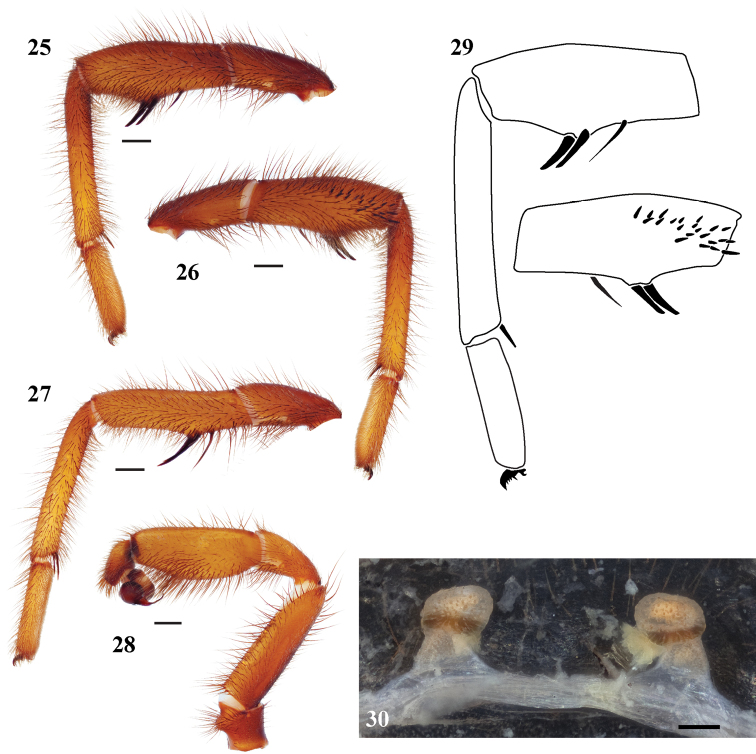
*Eucteniza relata* (O.P.-Cambridge, 1895) from Kingsville, Texas **25–29** male specimen **25** retrolateral aspect, leg I [832050] **26** prolateral aspect, leg I [832046] **27** retrolateral aspect, leg II [832052] **28** retrolateral aspect, pedipalp [832054] **29** line drawings, leg I retrolateral and prolateral (tibia) aspects **30** cleared spermathecae, scale bar = 0.1mm [832096].

#### Description.

*Eucteniza relata* described by O.P.-Cambridge (1895). Synonyms (*Eucteniza rex* and *Eucteniza stolida*) described by Chamberlin (1940), Gertsch and Mulaik 1940, and Bond and Opell (2002). The holotype for *Eucteniza rex* was illustrated by Bond and Opell (2002).

**Variation (males, 6).** Cl 8.07-11.53, 9.59±0.47; Cw 7.26-9.76, 8.28±0.37; STRl 4.73-5.98, 5.3±0.19; STRw 4.34-6.01, 5.06±0.26; LBw 1.18-1.56, 1.4±0.06; LBl 0.78-1.14, 0.98±0.05; leg I: 7.39-9.3, 8.24±0.28; 3.86-5.09, 4.44±0.18; 5.04-6.38, 5.41±0.22; 5.55-7.23, 6.38±0.26; 2.78-3.99, 3.34±0.18; leg IV: 7.74-9.88, 8.58±0.32; 3.98-5.15, 4.41±0.16; 5.98-7.93, 6.67±0.3; 6.47-9.02, 7.62±0.36; 3.64-5.31, 4.49±0.27; PTl 4.19-5.31, 4.64±0.16; PTw 1.87-2.25, 2.06±0.05; Bl 1.54-2.45, 1.89±0.13; TSp 4-21, 9.33±2.54; TSr 0-0, 0±0; TSrd 0-0, 0±0;

**Variation (females, 6).** Cl 7.55-10.01, 8.61±0.45; Cw 6.39-7.94, 7.31±0.31; STRl 4.4-5.89, 4.98±0.27; STRw 4.13-5.23, 4.68±0.21; LBw 1.39-1.98, 1.62±0.11; LBl 0.96-1.39, 1.16±0.08; Leg I: 13.54-20.45, 17.11±1.23; ANTd 6-8, 7.2±0.37; PTLs 45-80, 63.6±7.02; TBs 16-36, 23.8±3.29.

#### Material examined.

**Mexico: Guerrero:** Taxco, 18.5351, -99.6098^6^, 1621m, V Roth, W Gertsch 29.vii.1956 [EU030, 1♂, AMNH]; **Nuevo Leon:** Cerro Potosi, Galeana, 24.8811, -100.2327^5^, 3713m, S & J Peck 26.vi.1969 [EU061, 1♀, AMNH]; 20mi W of Linares, 24.8666, -99.8848^6^, 1894m, S Mulaik 1.ix.1956 [EU074, 2♀, AMNH]; **Tamaulipas:** Conrado Castillo, 23.9500, -99.4667^6^, 1955m, P Sprouse v.1980 [EU003, 1♀, AMNH]; Antiguo Morelos, Mexico, 22.5500, -99.0833^5^, 205m, JA Beatty 21.vi.1963 [EU036, 1♂, AMNH]; Tampico, Mexico, 22.3000, -97.8500^5^, 10m, Ekhomb 1942 [EU013, 1♀, AMNH]. **United States: Texas: Atascosa Co.:** 28.8333, -98.5000^7^, 106m, C Rutherford 31.xii.1936 [EU062, 1♀, AMNH]; Jourdanton, 28.9178, -98.5461^3^, 140m, C Rutherford [EU046, 1♀, AMNH]; Jourdanton, 28.9178, -98.5461^3^, 140m, C Rutherford 27.xi.1935 [EU058, 1♀, AMNH]; **Bastrop Co.:** Bastrop State Park, 30.1122, -97.2606^3^, 168m, B Hunsacker 26.iii.1958 [EU029, 1♀, AMNH]; Little Sandy Creek 10mi NW of Bastrop, 30.2601, -97.3555^3^, 125m, B Vogel 4.x.1971 [EU064, 1♂, AMNH]; **Bell Co.:** Temple, 31.0981, -97.3427^6^, 221m, M Hatley x.2006 [EU084, 1♂, TAMU]; **Bexar Co.:** San Antonio, 29.4239, -98.4933^7^, 198m, L Griffith 15.xii.1939 [EU049, 1♂, AMNH]; San Antonio, 29.424, -98.4833^6^, 199m, L Gonzales 10.xii.1987 [EU088, 1♂, TAMU]; San Antonio, 29.424, -98.4833^6^, 199m, L Monney 20.x.1989 [EU089, 1♂, TAMU]; San Antonio, 29.424, -98.4833^6^, 199m, R Uasquez 15.xi.1992 [EU090, 1♂, TAMU]; **Dimmit Co.:** 3mi NW of Catarina, 28.3747, -99.0107^1^, 166, BE Hendrixson, M Capes, M Roberts 12.iii.2000 [EU092, 1♀, AUMNH]; **Duval Co.:** 4mi E of Freer, 27.8828, -98.5494^5^, 186m, EG Riley 11.x.2003 [EU085, 1♂, TAMU]; **Hidalgo Co.:** Mission, 26.2156, -98.325^3^, 43m [EU007, 1♀, AMNH]; Edinburg, 26.3014, -98.1631^3^, 29m, S Mulaik [EU026, 1♀, AMNH]; Edinburg, 26.3014, -98.1631^3^, 29m, S Mulaik [EU033, 1♀, AMNH]; Edinburg, 26.3014, -98.1631^3^, 29m, S Mulaik 1.iii.1936 [EU027, 1♀, AMNH]; Edinburg, 26.3014, -98.1631^3^, 29m, S Mulaik 1.v.1937 [EU048, 1♀, AMNH]; Edinburg, 26.3014, -98.1631^3^, 29m, S Mulaik 27.ii.1939 [EU050, 1♀, 1 juv, AMNH]; Edinburg, 26.3014, -98.1631^3^, 29m, S Mulaik 1.iii.1938 [EU032, 2 juv, AMNH]; Edinburg, 26.3014, -98.1631^3^, 29m, D Mulaik 5.vi.1939 [EU039, 1♂, AMNH];Edinburg, 26.3014, -98.1631^3^, 29m, 24.xii.1949 [EU040, 1♀, AMNH]; Edinburg, 26.3014, -98.1631^3^, 29m, S Mulaik 15.iv.1936 [EU042, 1♀, AMNH]; Edinburg, 26.3014, -98.1631^3^, 29m, S Mulaik 31.xii.1934 [EU044, 069, 2♀, AMNH]; **Houston Co.:** Old Tyler Rd near Ratcliff, 31.3914, -95.1394^6^, 122m, SFA Student 23.ix.1968 [EU022, 1♀, AMNH]; **Kendall Co.:** Boerne, 39.7945, -98.7319^6^, 429m, ND Masters 20.ix.1994 [EU086, 1♀, TAMU]; **Kennedy Co.:** 50mi NW of Edinburg, 26.8796, -98.6642^7^, 154m, 24.ii.1949 [EU023, 1♀, AMNH]; **Kerr Co.:** Raven Ranch, 30.0666, -99.3333^7^, 546m, J McHenry 27.vi.1941 [EU053, 1♀, AMNH]; **Kleberg Co.:** Kingsville, 27.5156, -97.8558^6^, 18m, [EU063, 1♂, AMNH, EU037, 1♂, AMNH]; Kingsville, 27.5156, -97.8558^6^, 18m, JC Cross 31.xii.1944 [EU065, 1♂, AMNH]; Kingsville, 27.5156, -97.8558^6^, 18m, JC Cross 1.xi.1947 [EU066, 1♂, AMNH]; **LaSalle Co.:** 9mi W of Fowlerton on Hwy 97, 28.4534, -98.9598^4^, 110m, EG Riley 11.x.2003 [EU099, 1♂, AUMNH]; **Midland Co.:** Midland, 31.9973, -102.0779^6^, 848m, M Dilley Summer 2002 [EU082, 1♂, AUMNH]; **Nueces Co.:** Robstown, 27.7900, -97.6686^6^, 21m, 14.x.1968 [EU035, 1♂, AMNH]; Robstown, 27.7900, -97.6686^6^, 21m, Richard 10.ix.1968 [EU055, 1♂, AMNH]; **Sabine Co.:** 9mi E of Hemphill, “Beech Bottom”, 31.3405, -93.6948^5^, 58m, Anderson, Morris 15.xi.1985 [EU087, 1♀, 2♂, TAMU]; **San Patricio Co.:** 8mi NE of Sinton, 28.087, -97.3741^5^, 10m, HE Laughlin 15.x.1959 [EU019, 1♂, AMNH]; **Starr Co.:** 26.5666, -98.7333^7^, 135m, V Wilder 25.ix.1940 [EU043, 1♀, AMNH]; Near Hwy 83, 2.5mi W of Sullivan City, 26.2894, -98.5938^4^, 58m, WR Icenogle 6.ix.1974 [EU067, 1♂, AUMNH]; **Sutton Co.:** 30.5166, -100.6333^7^, 647m, L Pierce 17.ii.1973 [EU005, 1♂, AMNH]; **Travis Co.:** SRD University of Texas Campus, 30.2918, -97.7385^3^, 168m, WH McAlister 10.ii.1956 [EU006, 1♀, AMNH]; 5mi E of Austin, 30.3392, -97.5926^6^, 183m, WF Blair 21.i.1957 [EU020, 1♂, AMNH]; Austin, 30.2669, -97.7428^3^, 153m, 1.iii.2005 [EU034, 1♀, AMNH]; Austin, 30.2669, -97.7428^3^, 153m, Casteel 3.xii.1945 [EU041, 1♂, AMNH]; Austin, 30.2669, -97.7428^3^, 153m, 29.xi.1945 [EU054, 1♀, AMNH]; Austin, 30.2669, -97.7428^3^, 153m, 31.xii.1971 [EU070, 1♀, 2♂, AMNH]; Austin Caverns, 30.2969, -97.7743^6^, 152m, B Russel 3.x.1964 [EU072, 1♂, AMNH]; Austin, 30.2669, -97.7428^3^, 153m, 31.xii.2003 [EU076 Paratype, 1♀, AMNH]; Austin, 30.2671 Austin, 30.2671^6^, 148m, J Heskett xii.2003 [EU083, 1♂, TAMU]; Austin, 30.2671 Austin, 30.2671^6^, 148m, 1.vii.1983 [UMM460, 1♂, AMNH]; **Ward Co.:** 5mi N of Monahans, 31.6352, -102.9727^5^, 808m, J Brown 7.xi.1993 [EU051, 1♂, AMNH]; **Webb Co.:** Near Hwy 83, 1.8mi N of jct w/Hwy 35, 27.7889, -99.4557^1^, 213m, JE Bond 7.viii.1997 [EU107, 1♀, AUMNH]; Near Hwy 83, 1.8mi N of jct w/Hwy 35, 27.7889, -99.4557^1^, 213m, JE Bond 7.viii.1997 [EU057, 077, 078, 079, 4♀, AMNH]; Near Hwy 83, 1.8mi N of jct w/Hwy 35, 27.7889, -99.4557^1^, 213m, WR Icenogle 8.ix.1974 [EU016, 1♀, AUMNH]; Near Hwy 83, 1.8mi N of jct w/Hwy 35, 27.7889, -99.4557^1^, 213m, WR Icenogle 8.ix.1974 [EU059, 1♀, AUMNH]; **Zapata Co.:** 35mi NW of Rio Grande City on Rt 83, 26.7299, -99.1124^3^, 350m, S Mulaik [EU045, 1♀, 2 juv, AMNH].

#### Distribution.

Widely distributed throughout Texas and northern/central Mexico (Fig. 1).

#### Remarks.

Without doubt this species, as circumscribed herein, represents multiple species, likely cryptic. Until additional data are available (e.g., molecules) we have chosen to be conservative and strictly apply a morphological species concept as described above.

### Eucteniza diablo sp. n.

http://zoobank.org/C250C475-64A2-4568-A2ED-C97F9DF8E9E6

http://species-id.net/wiki/Eucteniza_diablo

**Figures 31–36.**
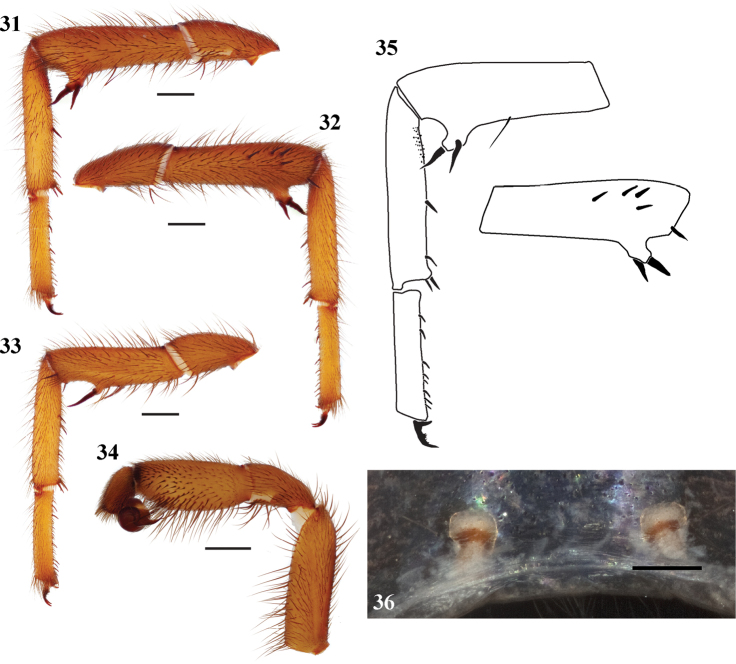
*Eucteniza diablo* sp. n. from Mexico, Baja California Surmale holotype and female paratype **31–35** male specimen **31** retrolateral aspect, leg I [832074] **32** prolateral aspect, leg I [832070] **33** prolateral aspect, right leg II [832076] **34** retrolateral aspect, pedipalp [832078] **35** line drawings, leg I retrolateral and prolateral (tibia) aspects **36** cleared spermathecae, scale bar = 0.1mm [832098].

‘The Baja California Trapdoor Spider’ Figs 1 , 31–36

#### Type material.

Male holotype (EU081), from Mexico, Baja California Sur, 3.2km S of La Paz, 24.103, -110.307^5^, 58m 10.viii.1966; additional male paratypes (EU095) from Baja California Sur, Mexico, El Sombrero Trailer Park in La Paz, 24.1331, -110.2998^5^, 47m 3.vii.1968, coll. M. Bentzien; additional female paratype (EU102) from Mexico, Baja California Sur, 2mi S of Santa Rita, 24.572, -111.4364^4^, 36m 16.xi.1968, coll. E.L. Sleeper and F.J. Moore. Male holotype and male and female paratypes deposited in AMNH.

#### Etymology.

The specific epithet is a noun taken in apposition and is in reference to the highest peak on the Baja Peninsula, “Picacho del Diablo”.

#### Diagnosis.

Male *Eucteniza diablo* specimens can be differentiated from all other species in the genus by having, in combination, leg I tibia megaspines borne on a mid-ventral apophysis, small microspines on the ventral distal aspect of metatarsus, short ventral spines on tarsus I, and a curved tarsus III (Figs 31, 32, 35). Similar to *Eucteniza zapatista* but lacking retrolateral spines on male palpal tibia (Fig. 34).

#### Description of male holotype.

*Specimen preparation and condition*. Specimen preserved in 80% EtOH. Multiple legs removed, stored in vial with specimen. Coloration faded. *General coloration*. Carapace dark reddish brown 2.5YR 3/4. Abdomen yellowish red 5YR 4/6. *Cephalothorax*. Carapace 6.31 long, 5.15 wide, glabrous, no distinct fringe, pars cephalica moderately elevated. Foveal groove strongly procurved, deep and shelf-like. Eyes without tubercle. AER slightly procurved, PER straight. AME, PME subequal, AME slightly smaller and PLE reduced in size, very small. Sternum with light setae, STRl 3.56, STRw 3.12. Posterior sternal sigilla in center, not contiguous, irregular shape, medial anterior sigilla pair inset, anterior pair marginal. Chelicerae with anterior tooth row comprising 5 large teeth, posterior margin with single row of 7 teeth. Palpal endites with cuspules distributed across entire face, labium with 14 cuspules, LBw 1.12, LBl 0.81. Rastellum consists of 8 spines. *Abdomen*. Moderate to dense setation. *Legs*. Leg I: 5.65, 2.50, 4.10, 3.95, 2.70; leg IV: 5.75, 2.50, 4.75, 5.00, 3.05. Very light scopulae on legs I, II. Tarsus leg III slightly curved, microspines on metatarsus proximal to junction with tibia; small spines on tarsus I ventral surface (Figs 31, 32, 35); TSp 4, TSr 0, TSrd 0. *Pedipalp*. PTw 1.28, PTl 2.50, Bl 1.31 (Fig. 34).

#### Variation.

Known only from the type specimens.

#### Description of female paratype.

*Specimen preparation and condition*. Specimen preserved in same manner as male holotype. *Color*. Carapace dark red 2.5YR 3/6. Spinnerets light yellow. *Cephalothorax*. Carapace 6.3 long, 5.9 wide, glabrous. Lacks fringe. Foveal groove deep and procurved. Tubercle absent. AER very slightly procurved, PER straight. AME, PME subequal. Sternum moderately setose, STRl 4.40, STRw 3.65. Posterior sigilla large and nearly contiguous, medial anterior sigilla relatively large and positioned more towards center. Chelicerae anterior tooth row armed with 6 teeth with posterior margin comprising 4 teeth. Palpal cuspules numerous and widespread across endites; labium with 12 cuspules, LBw 1.14, LBl 0.88. Rastellum consists of 10 spines positioned on a mound. *Walking legs*. Leg I 14.43 long. Tarsus I with 12 widely scattered trichobothria. Legs I, II with heavy, asymmetric scopulae. PTLs 32, TBs 15. Preening combs absent. Spermathecae simple bulb (Fig. 36).

#### Additional material examined.

**Mexico: Baja California Sur:** 27.3mi S Santa Rita, 24.2548, -111.2376^5^, 29m, SC Williams, J Bigelow, M Bentzien 27.vii.1968 [EU097, 1♂, AMNH]; La Paz city limits, 24.1331, -110.2998^5^, 8m, SC Williams, MA Cazier, M Bentzian, WK Fox, J Bigelow 13.vii.1968 [EU094, 1♂, AMNH].

#### Distribution.

Known from the La Paz municipality of Baja California Sur, Mexico (Fig. 1).

### Eucteniza cabowabo sp. n.

http://zoobank.org/CE5B7237-6056-4899-BA7B-67CC0AE7F4AB

http://species-id.net/wiki/Eucteniza_cabowabo

**Figures 37–42.**
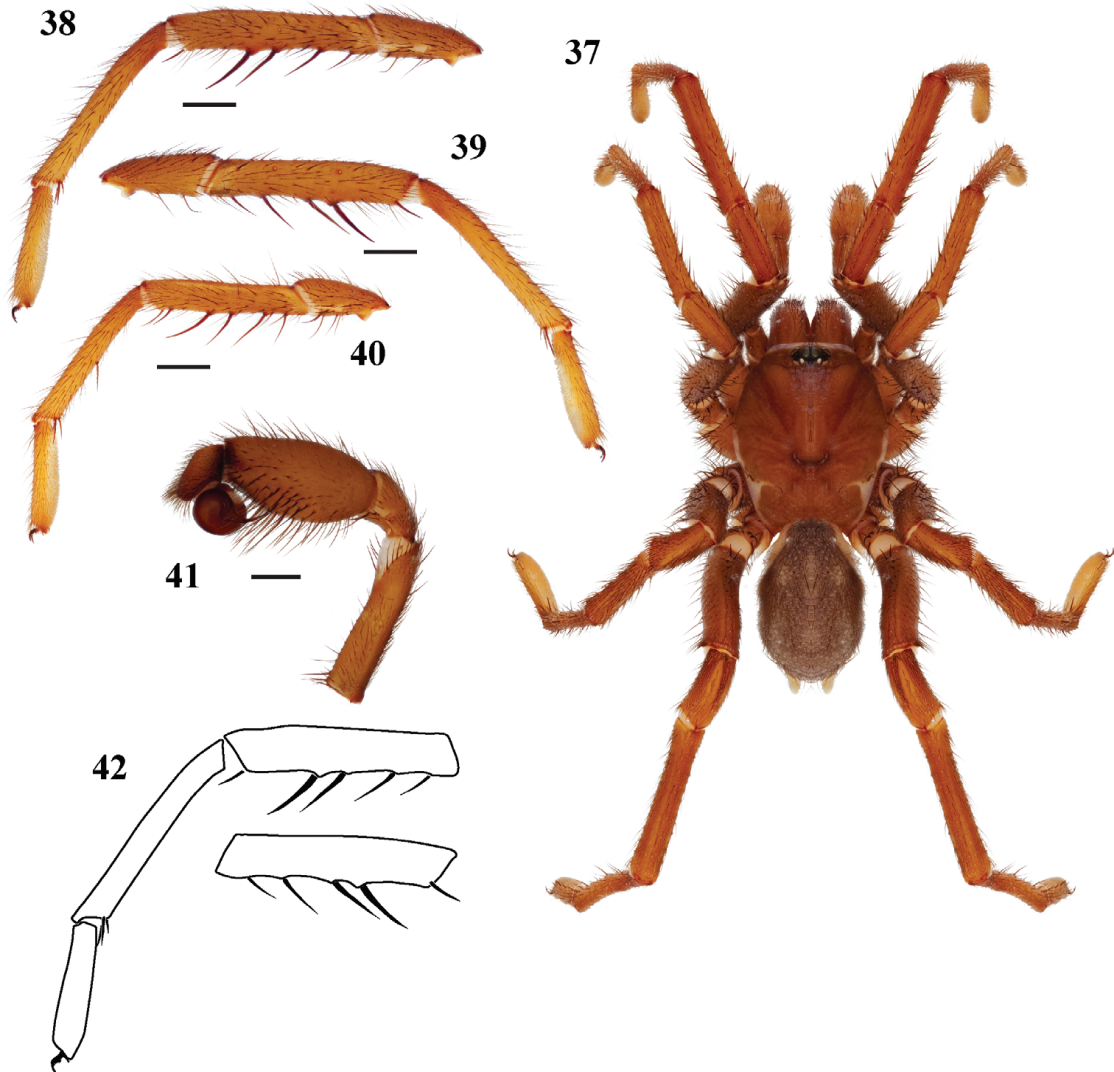
*Eucteniza cabowabo* sp. n. from Mexico, Baja California Surmale holotype **37** habitus [832094] **38** retrolateral aspect, leg I [832064] **39** prolateral aspect, leg I [832060] **40** retrolateral aspect, leg II [832066] **41** retrolateral aspect, pedipalp [832068] **42** line drawings, leg I retrolateral and prolateral (tibia) aspects.

‘The Cabo Wabo Trapdoor Spider’ Figs 1 , 37–42

#### Type material.

Male holotype and female paratype (EU096), from Baja California Sur, Mexico, 8mi SE of La Paz, 24.0338, -110.2331^4^, 287m, coll. E. L. Sleeper, F. J. Moore 13.x.1968. Male holotype and female paratype deposited in AMNH.

#### Etymology.

The specific epithet is a noun taken in apposition inspired by Sammy Hagar’s club and restaurant, Cabo Wabo, in Cabo San Lucas.

#### Diagnosis.

Male *Eucteniza cabowabo* specimens differ from all other *Eucteniza* specimens by having a very slender leg I tibia and metatarsus with thin ventral megaspines (Figs 3–40, 42); PME’s reduced in size. The single *Eucteniza cabowabo* female paratype lacks PME’s; due to the lack of specimens it is not clear whether this is a diagnostic feature or the specimen is aberrant.

#### Description of male holotype.

*Specimen preparation and condition*. Specimen preserved in 70% EtOH. Pedipalp, leg I, and leg II left side removed, stored in vial with specimen. Coloration faded. *General coloration*. Carapace dark reddish brown 2.5YR 2.5/4 (Fig. 37). Abdomen very dusky red 2.5YR 2.5/2. *Cephalothorax*. Carapace 5.25 long, 4.19 wide, glabrous, lacks fringe, pars cephalica low. Foveal groove deep and procurved. Tubercle absent. AER slightly procurved, PER slightly recurved. AME slightly larger in diameter than PME. Sternum moderately setose, STRl 3.04, STRw 2.48. Posterior sternal sigilla large, irregularly shaped, and contiguous, medial pair anterior sigilla more centrally positioned, irregularly shaped. Chelicerae with anterior tooth row comprising 6 teeth, posterior margin with single straight row of 4 teeth. Lacks palpal and labium cuspules, LBw 0.83, LBl 0.45. Rastellum consists of 8 spines. *Abdomen*. Moderately setose. *Legs*. Leg I: 5.44, 2.38, 3.88, 4.19, 2.31; leg IV: 5.69, 2.38, 4.69, 4.75, 3.19. Light scopulae on legs I, II, III, IV. Tarsus with 10 trichobothria, widely spread. Leg I spination; TSp 3, TSr 0, TSrd 0 (Figs 38, 39, 42). *Pedipalp*. PTw 1.36, PTl 2.60, Bl 1.35 (Fig. 41).

#### Variation.

Known only from the type specimens and one other male.

#### Description of female paratype.

*Specimen preparation and condition*. Specimen preserved in same manner as male holotype. *Color*. Carapace dark red 2.5YR 3/6. Spinnerets light yellow. *Cephalothorax*. Carapace 7.0 long, 4.81 wide, glabrous. Lacks fringe. Foveal groove deep and procurved. Tubercle absent. AER very slightly procurved, PER straight. PME absent. Sternum moderately setose, STRl 4.60, STRw 3.20. Posterior sigilla large and nearly contiguous, medial anterior sigilla relatively large and positioned more towards center. Chelicerae anterior tooth row armed with 6 teeth with posterior margin comprising 4 teeth. Palpal cuspules numerous and widespread across endites; labium with 12 cuspules, LBw 1.26, LBl 0.77. Rastellum consists of 14 spines positioned on a mound. *Walking legs*. Leg I 12.40 long. Tarsus I with 12 widely scattered trichobothria. Legs I, II with heavy, asymmetric scopulae. PTLs 25, TBs 16. Preening combs absent. Spermathecae not with specimen, presumed lost.

#### Variation.

Known only from the female paratype specimen.

#### Additional material examined.

**Mexico: Baja California Sur:** 6mi E of Cabo San Lucus, 22.9248, -109.8187^4^, 12m, H Ridgeway 13.i.1974 [EU093, 1♂, AMNH].

#### Distribution.

Known from La Paz and Los Cabos municipalities of Baja California Sur, Mexico (Fig. 1).

### Eucteniza huasteca sp. n.

http://zoobank.org/2E8ECBC1-E7D7-469C-AEE1-975C8BD0520E

http://species-id.net/wiki/Eucteniza_huasteca

**Figures 43–47.**
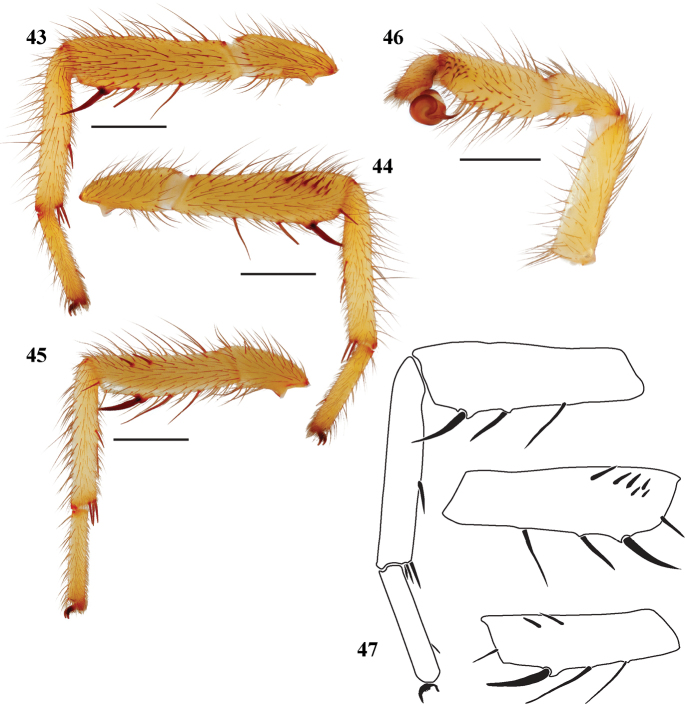
*Eucteniza huasteca* sp. n. from Nuevo Leon, Mexico male holotype **43** retrolateral aspect, leg I [832040] **44** prolateral aspect, leg I [832036] **45** retrolateral aspect, leg II [832042] **46** retrolateral aspect, pedipalp [832044] **47** line drawings, leg I retrolateral and prolateral (tibia) aspects; prolateral aspect tibia leg II.

‘The Huasteca Canyon Trapdoor Spider’ Figs 1 , 43–47

#### Type material.

Male holotype (EU052), from Nuevo Leon, Mexico, at La Huasteca Canyon, 3mi SW of Santa Catarina, 25.6544, -100.5075^4^, 1114m, coll. L. Malarat 11.viii.1978; deposited in AMNH.

#### Etymology.

The specific epithet is a noun taken in apposition and is in reference to the type locality.

#### Diagnosis.

Male *Eucteniza huasteca* type specimen differs from all other *Eucteniza* species on the basis of its very small size, very pale yellow coloration, and by having a distinct patch of spines on the distal aspect of the palpal tibia (Figs 43, 44, 47); other species are typically larger in size, darker in coloration, and lack similar palpal tibia spination.

#### Description of male holotype.

*Specimen preparation and condition*. Specimen preserved in 70% EtOH. Pedipalp, legs I, II removed, stored in vial with specimen. Coloration faded. *General coloration*. Carapace yellowish red 5YR 5/8. Abdomen reddish yellow 7.5YR 6/8. *Cephalothorax*. Carapace 3.48 long, 2.53 wide, glabrous with sparse posterior fringe, pars cephalica low. Foveal groove procurved. Eyes slightly elevated. AER straight, PER slightly recurved. AME and PME subequal. Sternum with long setae, STRl 2.06, STRw 1.50. Posterior sternal sigilla large and elongate, but separated, anterior pairs marginal, difficult to see. Chelicerae with anterior tooth row comprising 6 teeth, posterior margin with 3 small denticles. Palpal endites and labium lack cuspules, LBw 0.54, LBl 0.28. Rastellum consists of 6 spines. *Abdomen*. Long thin setae. *Legs*. Leg I: 3.09, 1.47, 2.22, 2.22, 1.34; leg IV: 3.13, 1.25, 3.03, 2.81, 1.47. Very light scopulae on legs I-II. Tarsus with 4 trichobothria. Leg I spination pattern; TSp 6, TSr 0, TSrd 0 (Figs 43, 44, 47); Leg II spination pattern Figs 45, 47. *Pedipalp*. PTw 0.56, PTl 1.41, Bl 0.73. Embolus slender (Fig. 46).

#### Variation.

Known only from the type specimen.

#### Description of female.

Known only from the male holotype specimen.

#### Distribution.

Known from Nueva Leon, Mexico, at La Huasteca Canyon (Fig. 1).

### Eucteniza zapatista sp. n.

http://zoobank.org/D1B109F8-8692-4CCC-BBA6-57FF7EDAAFE2

http://species-id.net/wiki/Eucteniza_zapatista

**Figures 48–52.**
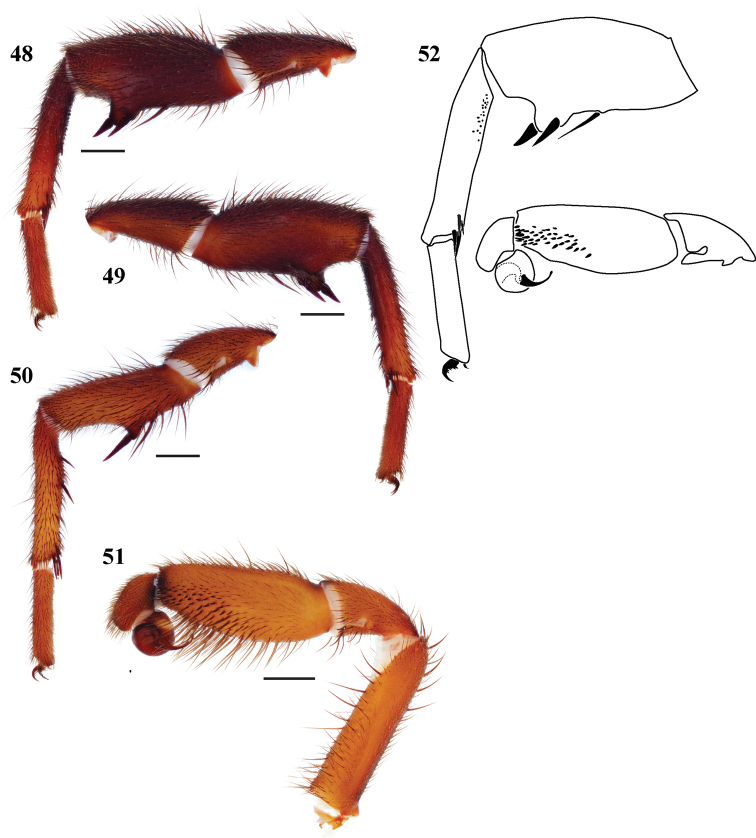
*Eucteniza zapatista* sp. n. from Paso de Cortes, Puebla, Mexico, male holotype **48** retrolateral aspect, leg I [832010] **49** prolateral aspect, leg I [832006] **50** retrolateral aspect, leg II [832012] **51** retrolateral aspect, pedipalp [832014] **52** line drawings, leg I retrolateral aspect; pedipalp, retrolateral aspect.

‘The Zapatista Trapdoor Spider’ Figs 1 , 48–52

#### Type material.

Male holotype (EU012), from Paso de Cortes, Puebla, Mexico, 19.1167, -98.7667^6^, 3000m, coll. C. Bolivar 18.vii.1943. Male holotype deposited in AMNH.

#### Etymology.

The specific epithet is a noun taken in apposition and is in reference to the common name used for the Mexican Liberation Army of the South (Ejército Libertador del Sur) led by Emiliano Zapata (1879-1919).

#### Diagnosis.

Male *Eucteniza zapatista* leg I morphology is similar to *Eucteniza diablo* however it lacks tarsal spines and has a more inflated or swollen tibia (Figs 48, 49, 52). Males can be further distinguished from all other species by having an extensive patch of spines on the retrolateral distal aspect of the palpal tibia (Figs 51, 52).

#### Description of male holotype.

*Specimen preparation and condition*. Specimen preserved in 70% EtOH. Pedipalp, legs I, II removed, stored in vial with specimen. Coloration faded. *General coloration*. Carapace dark reddish brown 2.5YR 3/4. Abdomen reddish black 2.5YR 2.5/1. *Cephalothorax*. Carapace 6.13 long and 5.56 wide, with dense fringe of black setae. Foveal groove deep and procurved. AME slightly elevated in center. AER and PER relatively straight. AME slightly larger in diameter than PME. Sternum moderately setose, STRl 3.56, STRw 3.24. Posterior sternal sigilla large, elongate and contiguous, medial pair of anterior sigilla moderate in size and inset, anterior pair small and marginal. Chelicerae with anterior tooth row comprising 9 teeth, posterior margin with patch of approximately 20 small denticles. Palpal endites with very small, evenly distributed, poorly defined cuspules, LBw 1.26, LBl 0.75. Rastellum consists of 6 spines on a mound. *Legs*. Leg I 5.69, 3.00, 3.76, 3.68, 2.13; leg IV: 5.31, 2.80, 4.36, 4.80, 2.60. Very light tarsal scopulae on legs I, II, III. Leg I tibia with 2 very stout, short megaspines with prominent base; TSp 0, TSr 0, TSrd 0 (Figs 48, 49, 52); Leg II spination Fig. 50. *Pedipalp*. PTw 1.36, PTl 3.32, Bl 1.50. Embolus slightly flared tip; dense spine patch tibia distal retrolateral aspect (Figs 51, 52).

#### Variation.

Known only from the single type specimen.

#### Description of female.

Known only from the male holotype specimen.

#### Distribution.

Known only from the type locality, municipality of Puebla, Mexico (Fig. 1).

### Eucteniza chichimeca sp. n.

http://zoobank.org/123BAC44-C19D-4131-8A37-9D6E43E8D178

http://species-id.net/wiki/Eucteniza_chichimeca

**Figures 53–57.**
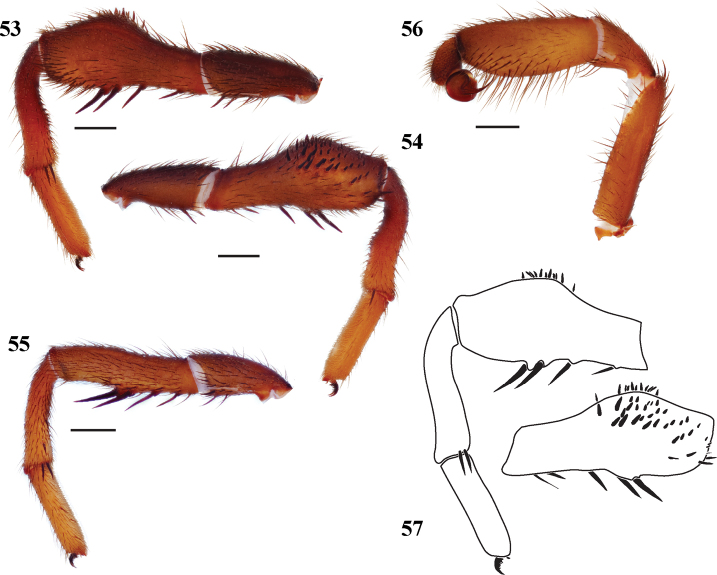
*Eucteniza chichimeca* sp. n. from Querétaro, Mexico, male holotype **53** retrolateral aspect, leg I [832000] **54** prolateral aspect, leg I [831996] **55** retrolateral aspect, leg II [832002] **56** retrolateral aspect, pedipalp [832004] **57** line drawings, leg I retrolateral and prolateral (tibia) aspects.

‘The Chichimeca Jonaz Trapdoor Spider’ Figs 1 , 53–57

#### Type material.

Male holotype (EU010), from Querétaro, Mexico, 20km N Pinal de Amoles, 21.15, -99.65^6^, 2227m, coll. W. Russell, J. Greer 5-6.vi.1971. Male holotype deposited in AMNH.

#### Etymology.

The specific epithet is a noun taken in apposition and refers to one of the groups of people that are indigenous to the area around the type locality, the Chichimeca Jonaz.

#### Diagnosis.

Male *Eucteniza chichimeca* specimens can be distinguished from all other *Eucteniza* species by virtue of having a tibia I that is swollen distal-dorsally and with numerous small prolateral spines (Figs 53, 54, 57).

#### Description of male holotype.

*Specimen preparation and condition*. Specimen preserved in 70% EtOH. Pedipalp, legs I, II removed, stored in vial with specimen. Color faded. *General coloration*. Carapace dark reddish brown 2.5YR 2.5/3. Abdomen black 5YR 2.5/1. *Cephalothorax*. Carapace 5.88 long, 4.88 wide, glabrous, without fringe, pars cephalica moderately elevated. Foveal groove procurved. AER straight. PER very slightly recurved. AME and PME subequal. Sternum lightly setose, STRl 3.25, STRw 2.66. Chelicerae with anterior tooth row comprising 7 teeth, posterior margin with patch of approximately 10 small denticles. Palpal endites and labium without cuspules, LBw 1.13, LBl 0.69. Rastellum consists of 6 spines. *Abdomen*. Lightly setose. *Legs*. Leg I: 5.10, 3.00, 3.90, 3.30, 2.35; leg IV: 5.10, 2.00, 4.00, 4.00, 2.60. Scopulae present on legs I, II, lighter on legs III, IV. Leg I spination; TSp >30, TSr 0, TSrd 0 (Figs 53, 54, 57); Leg II spination Fig. 55. *Pedipalp*. PTw 1.28, PTl 3.16, Bl 1.31 (Fig. 56).

#### Variation.

Known only from the type specimen.

#### Description of female.

Known only from the male holotype specimen.

#### Distribution.

Known from the type locality, Querretara, Mexico (Fig. 1).

### Eucteniza ronnewtoni sp. n.

http://zoobank.org/282930D5-1FEF-40EA-B479-EDBE8CEC24A0

http://species-id.net/wiki/Eucteniza_ronnewtoni

**Figures 58–63.**
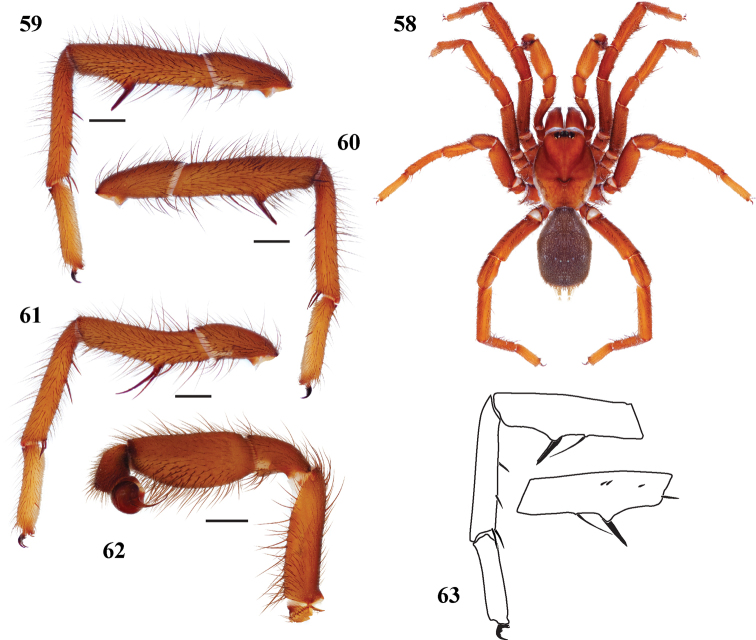
*Eucteniza ronnewtoni* sp. n. from Val Verde County, Texas, male holotype **58** habitus [832090] **59** retrolateral aspect, leg I [832020] **60** prolateral aspect, leg I [832016] **61** retrolateral aspect, leg II [832022] **62** retrolateral aspect, pedipalp [832024] **63** line drawings, leg I retrolateral and prolateral (tibia) aspects.

‘Ron Newton’s Pecos River Trapdoor Spider’ Figs 1 , 58–63

#### Type material.

Male holotype (EU015), from Val Verde County, Texas, on rocks at bridge on Pecos River, 29.7079, -101.351^4^, 396m, coll. J.A. Brubaker, F.J. Moore 2.ix.1968. Male holotype deposited in AMNH. Male paratype (EU080), from Sandy Canyon, 18 rd mi NE of Sauceda, 29.5550, -103.7933^1^, 1212m, coll. N.I. Platnick 4.x.2005. Male paratype deposited in AMNH.

#### Etymology.

The specific epithet is a patronym in honor of Dr. Ronald Newton, biologist and Texas native.

#### Diagnosis.

Male *Eucteniza ronnewtoni* specimens are similar in appearance to *Eucteniza relata* but have a more slender tibia I that has only a few small spines on the prolateral surface none of which are positioned distally (Figs 59, 60, 63).

#### Description of male holotype.

*Specimen preparation and condition*. Specimen preserved in 70% EtOH. Multiple legs removed, stored in vial with specimen. Color faded. *General coloration*. Carapace dark reddish brown 2.5YR 2.5/4. Abdomen very dark red 2.5YR 2.5/2. *Cephalothorax*. Carapace 5.44 long, 4.56 wide, glabrous, pars cephalica moderately elevated (Fig. 58). Fringe on posterior margin of light, black setae. Foveal groove deep, procurved. Tubercle absent. AER straight. PER slightly recurved. AME slightly larger in diameter than PME. Sternum lightly setose, STRl 3.41, STRw 2.91. Posterior sternal sigilla very large, not quite contiguous, medial anterior sigilla pair moderate in size, offset from margin, anterior pair not visible. Chelicerae with anterior tooth row comprising 5 large teeth, posterior margin with single row of 4 small teeth. Palpal endites with numerous cuspules across endite face, labium with 15 small cuspules, LBw 1.13, LBl 0.84. Rastellum consists of 8 small spines. *Abdomen*. Moderately setose. *Legs*. Leg I: 5.31, 2.40, 3.88, 3.68, 2.28; leg IV: 5.00, 1.88, 2.50, 3.28, 2.81. Light scopulae on legs I-IV. Tarsus I with wide band of 12 trichobothria. Leg I spination pattern; TSp 4, TSr 0, TSrd 0 (Figs 59, 60, 63); Leg II spination Fig. 61. *Pedipalp*. PTw 1.31, PTl 2.94, Bl 1.58 (Fig. 62). Embolus arises sharply from bulb and tapers quickly, slightly flared at tip.

#### Variation.

Known only from the type specimens.

#### Description of female.

Known only from the male type specimens.

#### Distribution.

Known from the type locality, Pecos River, Val Verde Co., Texas (Fig. 1).

### Eucteniza hidalgo sp. n.

http://zoobank.org/57B349E5-04EC-4D8E-A609-FEC414FB015B

http://species-id.net/wiki/Eucteniza_hidalgo

**Figures 64–68.**
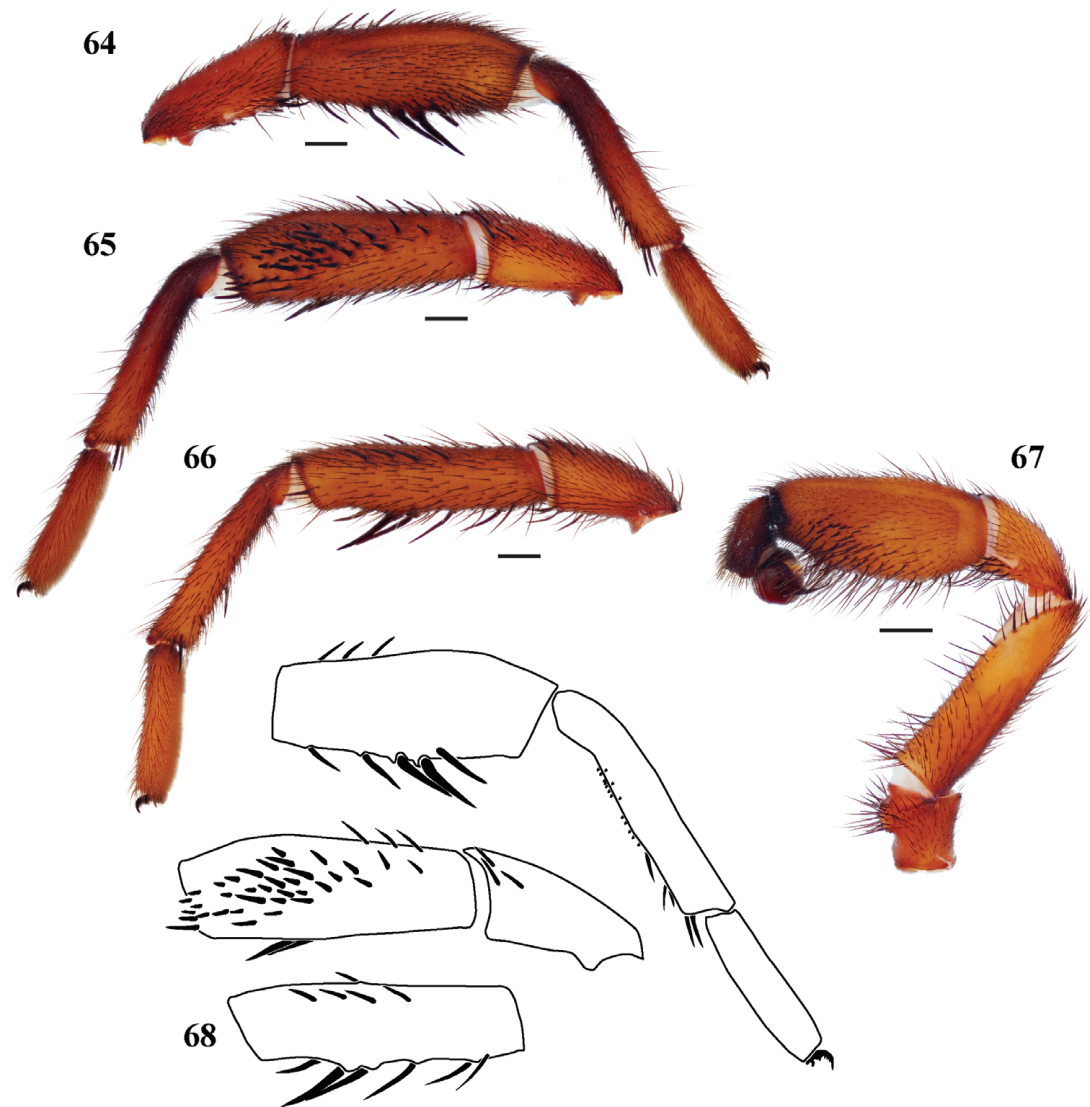
*Eucteniza hidalgo* sp. n. from Hidalgo, Mexico, male holotype **64** retrolateral aspect, right leg I [832026] **65** prolateral aspect, right leg I [832030] **66** prolateral aspect, right leg II [832032] **67** retrolateral aspect, pedipalp [832034] **68** line drawings, right leg I retrolateral and prolateral (tibia) aspects, right leg II prolateral aspect of tibia.

‘The Hidalgo Trapdoor Spider’ Figs 1 , 64–68

#### Type material.

Male holotype (EU047), from Hidalgo, Mexico. 20.6649, -99.0098^8^, 1509m, coll. T.C. Kaspar 2.viii.1973; deposited in AMNH.

#### Etymology.

The specific epithet is a noun taken in apposition and is in reference to the type locality in the state of Hidalgo, also used in reference to a person of noble or generous spirit.

#### Diagnosis.

The male *Eucteniza hidalgo* specimen differs from all other *Eucteniza* species by virtue of having an extensive prolateral tibia I spine patch, ventral metatarsus microspines, and a sub-dorsal row of spines on the prolateral surface of tibia II (Figs 64–68).

#### Description of male holotype.

*Specimen preparation and condition*. Specimen preserved in 70% EtOH. Multiple legs removed, stored in vial with specimen. Color faded. *General coloration*. Carapace dark reddish brown 2.5YR 2.5/4. Abdomen dark yellowish brown 10YR 4/6. *Cephalothorax*. Carapace 8.576 long, 7.263 wide, glabrous to sparsely setose posteriorly, pars cephalica very slightly elevated. Fringe on posterior margin with light, black setae. Foveal groove deep, procurved. Tubercle absent. AER very slightly procurved. PER slightly recurved. AME slightly larger in diameter than PME. Sternum setose, STRl 4.804, STRw 4.360. Posterior sternal sigilla very large, not quite contiguous, medial anterior sigilla pair moderate in size and marginal, anterior pair very small and marginal. Chelicerae with anterior tooth row comprising 8 large teeth, posterior margin with single row of 3 small teeth surrounded in small denticles. Palpal endites without cuspules across endite face, labium also without cuspules, LBw 1.457, LBl 0.938. Rastellum consists of 4 spines on a mound. *Abdomen*. Moderately setose. *Legs*. Leg I: 7.759, 4.371, 5.864, 5.384, 3.528; leg IV: 8.193, 4.059, 7.183, 7.257, 4.683. Light scopulae on legs I-II. Tarsus I with wide band of 10 trichobothria. Leg I spination pattern; TSp 34, TSr 0, TSrd 0 (Figs 64, 65, 68); Leg II spination pattern Figs 66, 68. *Pedipalp*. PTw 2.044, PTl 4.104, Bl 1.704. Embolus arises sharply from bulb and tapers quickly, slightly geniculate at tip (Fig. 67).

#### Variation.

Known only from the type specimen.

#### Description of female.

Known only from the male holotype specimen.

#### Distribution.

Known only from the type locality, Hidalgo, Mexico.

### Eucteniza golondrina sp. n.

http://zoobank.org/052429B4-EAE8-49D8-9FB4-C62128BAE9FE

http://species-id.net/wiki/Eucteniza_golondrina

**Figures 69–73.**
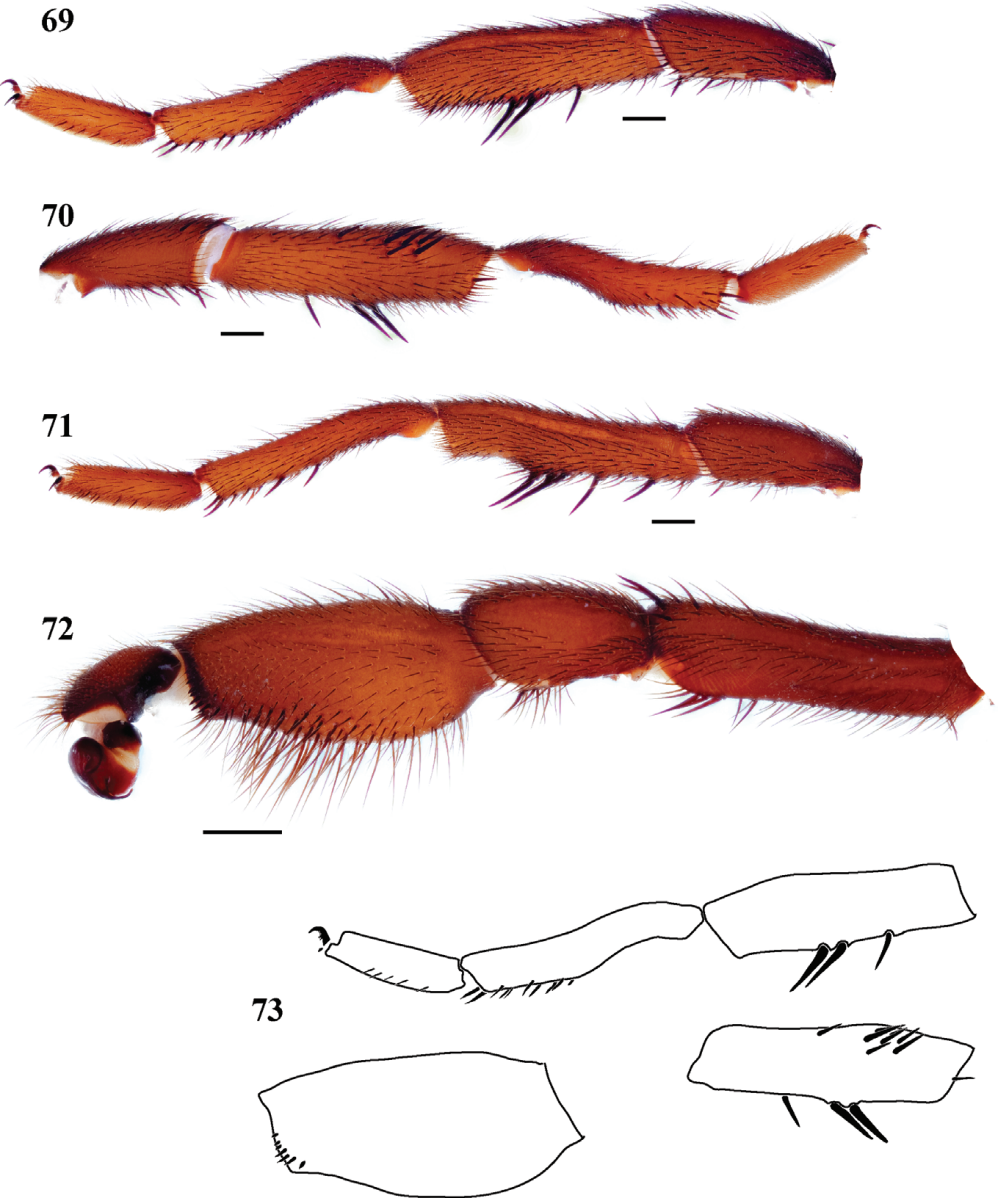
*Eucteniza golondrina* sp. n. from San Luis Potosí, Mexico, male holotype **69** retrolateral aspect, leg I [832104] **70** prolateral aspect, leg I [832100] **71** retrolateral aspect, leg II [832108] **72** retrolateral aspect, pedipalp [832106] **73** line drawings, leg I retrolateral and prolateral (tibia) aspects; retrolateral aspect, palpal tibia.

‘The Golondrina Trapdoor Spider’ Figs 1 , 69–73

#### Type material.

Male holotype (UMM117) from Sótano de las Golondrinas, Aquismón, San Luis Potosí, Mexico, 21.6263, -99.0292^4^, elev. 164m, coll. A. G. Grubbs xi.1987, deposited in AMNH.

#### Etymology.

The specific epithet is a noun taken in apposition and is in reference to the type locality Sótano de las Golondrinas (= Cave of Swallows).

#### Diagnosis.

The male *Eucteniza golondrina* type specimen differs from all other species of *Eucteniza* by virtue of a distinct leg I morphology that includes a unique group of distal spines on the ventral surface of metatarsus I (Figs 69, 70, 73); the palpal tibia of *Eucteniza golondrina* also has a retrolateral distal row of spines that is lacking in all other known species (Figs 72, 73).

#### Description of male holotype.

*Specimen preparation and condition*. Specimen preserved in 70% EtOH. Pedipalp, legs I, II removed, stored in vial with specimen. Color faded. *General coloration*. Carapace dark reddish brown 2.5YR 2.5/4. Abdomen black 10YR 2/1. *Cephalothorax*. Carapace 8.478 long, 7.437 wide, sparsely setose, few heavy setae posteriorly, pars cephalica slightly elevated. Fringe of sparse, heavy setae at posterior corners of carapace. Foveal groove deep, procurved. Tubercle absent. AER straight. PER straight. AME slightly larger in diameter than PME. Sternum moderately setose, STRl 4.695, STRw 3.874. Posterior sternal sigilla very large, not contiguous, tapering posteriorly, anterior sigilla pairs small and marginal. Chelicerae with anterior tooth row comprising 11 large teeth, posterior margin with single row of 14 small teeth. Palpal endites with few cuspules across endite face, labium lacking cuspules, LBw 1.377, LBl 0.758. Rastellum consists of 4 small spines. *Abdomen*. Moderately setose. *Legs*. Leg I: 8.354, 4.595, 6.006, 5.792, 3.034; leg IV: 8.586, 4.081, 6.970, 7.841, 4.161. Dense scopulae on legs I-II. Tarsus I with wide band of approximately 23 trichobothria. Leg I spination pattern; TSp 8, TSr 0, TSrd 0 (Figs 69, 70, 73); Leg II spination Fig. 71. *Pedipalp*. PTw 1.877, PTl 3.729, Bl 1.556. Embolus arises sharply from bulb and tapers quickly, geniculate at tip (Fig. 72); unique retrolateral distal row of tibial spines (Figs 72, 73).

#### Variation.

Known only from the type specimen.

#### Description of female.

Known only from the male holotype specimen.

#### Distribution.

Known only from the type locality, San Luis Potosí, Mexico.

### Eucteniza panchovillai sp. n.

http://zoobank.org/016EFBFF-8C22-45B6-B3DA-55864B5F6248

http://species-id.net/wiki/Eucteniza_panchovillai

**Figures 74, 75.**
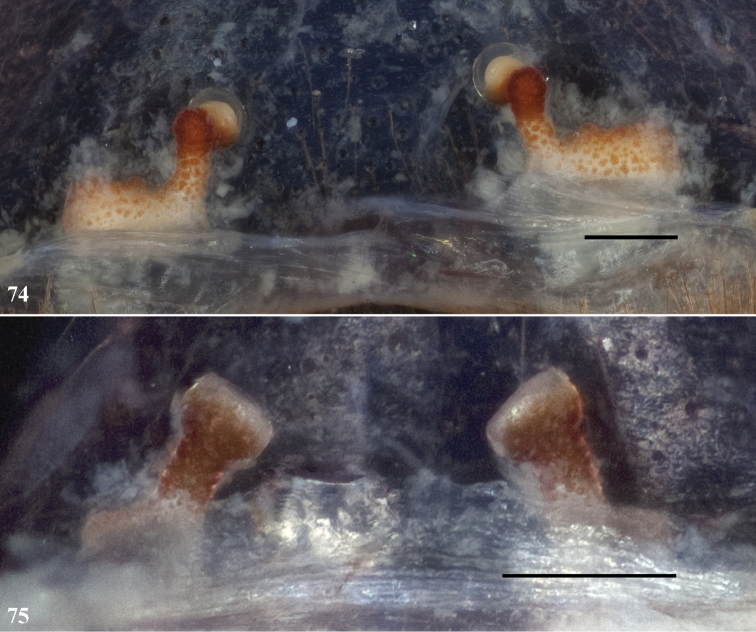
**74, 75** cleared spermathecae, scale bar = 0.1mm **74** *Eucteniza panchovillai* sp. n. female holotype from Durango, Mexico [832095] **75** *Eucteniza rosalia* sp. n. female holotype from Baja California Sur, Mexico [832097].

‘Pancho Villa’s Trapdoor Spider’ Figs 1 , 74

#### Type material.

Female holotype (EU060) and paratype (EU068), from San Juan del Rio, Durango, Mexico, 24.7833, -104.4500^5^, 1789m, coll. W. Gertsch 1.viii.1947. Female holotype deposited in AMNH.

#### Etymology.

The specific epithet is a patronym named for Mexican historical figure Pancho Villa.

#### Diagnosis.

Female specimens of *Eucteniza panchovillai* can be distinguished from all other known species by having spermathecae that comprise a long lateral extension and a slender stalk that curves distally into a small terminal bulb; all other species have shorter thicker stalks that do not curve distally and terminate in a larger bulb that exceeds the stalk diameter (Fig. 74).

#### Description of female holotype.

*Specimen preparation and condition*. Specimen preserved in 70% EtOH. Color faded. *Color*. Carapace yellowish red 5YR 4/6. Abdomen yellowish brown 10YR 5/6. *Cephalothorax*. Carapace 8.827 long, 8.064 wide, sparsely setose. Fringe absent. Foveal groove deep and procurved. Tubercle absent. AER procurved, PER slightly recurved. AME slightly larger in diameter than PME. Sternum moderately setose, STRl 5.364, STRw 5.318. Posterior sigilla large and nearly contiguous and irregular in shape, medial pair of anterior sigilla moderate in size and inset, nearly contiguous with posterior pair, anterior pair small and marginal. Chelicerae with anterior tooth row armed with 7 teeth with posterior margin comprising a long patch of many small denticles. Palpal cuspules numerous and widespread across endites; labium with 7 cuspules, LBw 2.033, LBl 1.446. Rastellum consists of 9 spines positioned on a small mound. *Walking legs*. Leg I 20.557 long. Tarsus I with 8 trichobothria clustered proximally. Legs I, II with dense scopulae. PTLs 64, TBs 19. Preening combs present. Spermathecae with enlarged laterally extended base, curved distally, terminal bulb width subequal to stalk (Fig. 74).

#### Variation

**(3).** Cl 8.01-9.87, 9.07±0.31; Cw 7.06-8.59, 8.12±0.27; STRl 4.52-5.8, 5.37±0.22; STRw 4.55-5.58, 5.24±0.19; LBw 1.66-2.25, 1.92±0.11; LBl 1.17-1.54, 1.31±0.07; Leg I: 18.61-21.79, 20.47±0.56; ANTd 7-9, 8.2±0.37; PTLs 25-38, 32±2.43; TBs 12-22, 15.6±1.94.

#### Description of male.

Known only from the female type specimens.

#### Additional material examined.

**Mexico: Durango:** San Juan del Rio, 24.7833, -104.4500^5^, 1789m, W Gertsch 1.viii.1947 [EU060, 068, 4♀, AMNH].

#### Distribution.

Known only from the type locality, Durango, Mexico.

### Eucteniza rosalia sp. n.

http://zoobank.org/8DE1B8F8-0C9F-4D82-A81A-1700C90549B4

http://species-id.net/wiki/Eucteniza_rosalia

‘The Río de Santa Rosalía Trapdoor spider’ Figs 1 , 75

#### Type material.

Female holotype (EU101), Mulegé, Baja California Sur, Mexico, 26.8905, -111.981^5^, 10m, coll. V. Roth 26.i.1965, deposited in AMNH.

#### Etymology.

The specific epithet is a noun taken in apposition and is in reference to the Río de Santa Rosalía near the type locality.

#### Diagnosis.

*Eucteniza rosalia* can be distinguished from other known Baja California taxa for which females are known by having a pronounced spermathecal lateral base extension by having a distally squared bulb (Fig. 75) as opposed to rounded. The spermathecal stalk in *Eucteniza diablo* is noticeably shorter and lacks a distinct lateral basal extension (Fig. 36).

#### Description of female holotype.

*Specimen preparation and condition*. Specimen preserved in 70% EtOH; color likely faded. *Color*. Carapace dark reddish brown 5YR 3/4. Abdomen dark yellowish brown 10YR 4/4. *Cephalothorax*. Carapace 5.332 long, 4.666 wide, sparsely setose. Fringe absent. Foveal groove deep and procurved. Tubercle absent. AER procurved, PER recurved. AME very slightly larger in diameter than PME. Sternum moderately setose, STRl 3.124, STRw 2.969. Posterior sigilla large and not contiguous, medial pair of anterior sigilla moderate in size and inset, anterior pair small and marginal. Chelicerae anterior tooth row armed with 6 teeth with posterior margin comprising a row of 9 small denticles. Palpal cuspules numerous and widespread across endites; labium with 11 cuspules, LBw 1.033, LBl 0.855. Rastellum consists of 7 spines positioned on a small mound. *Walking legs*. Leg I 11.219 long. Tarsus I with 10 trichobothria clustered proximally. Legs I, II with dense scopulae. PTLs 20, TBs 12. Preening combs absent. Spermathecae with moderately sized lateral base, terminal bulb square (Fig. 75).

#### Variation.

Known only from type specimen.

#### Description of male.

Known only from the female type specimens.

#### Distribution.

Known only from the type locality, Baja California Sur, Mexico.

## Supplementary Material

XML Treatment for Euctenizidae

XML Treatment for Euctenizinae

XML Treatment for Eucteniza

XML Treatment for Eucteniza mexicana

XML Treatment for Eucteniza caprica

XML Treatment for Eucteniza coylei

XML Treatment for Eucteniza relata

XML Treatment for Eucteniza diablo

XML Treatment for Eucteniza cabowabo

XML Treatment for Eucteniza huasteca

XML Treatment for Eucteniza zapatista

XML Treatment for Eucteniza chichimeca

XML Treatment for Eucteniza ronnewtoni

XML Treatment for Eucteniza hidalgo

XML Treatment for Eucteniza golondrina

XML Treatment for Eucteniza panchovillai

XML Treatment for Eucteniza rosalia

## Appendix

Locality data for *Eucteniza* specimens. (doi: 10.3897/zookeys.356.6227.app) File format: Microsoft Excel comma delimited (.csv).

**Explanation note:** Locality data for *Eucteniza* specimens examined over the course of this study and listed in material examined section that accompanies each species.
